# Supplementary material for: Growth dynamics of Escherichia coli cells on a surface having AgNbO3 antimicrobial particles
Source: PLoS One. 2024 Aug 19;19(8):e0305315. doi: 10.1371/journal.pone.0305315 (PMC11332949; doi:10.1371/journal.pone.0305315)
Supplement: S1 Appendix — (DOCX) [file pone.0305315.s001.docx]

# **S1 Appendix. Measuring solid phase growth rate**

The growth rate of microbial cells within a microcolony on an agar plate was determined through the following steps for a given microbial species (repeated across multiple microbial species):

1. Prepare starting cell suspension with a nominal concentration of 10^5^ CFU/mL.

*2.* Dispense 1 µL of the cell suspension on each one of five agar gel plates (P#0, P#1, P#2, P#3, and P#4) and allow to dry in air for 15 min.

3. Incubate the plates P#1, P#2, P#3, P#4 at 37^o^ C, respectively, for 2, 3, 4, and 6 h.

4. For P#0, harvest the microbial content of the spot (all microcolonies in) by a swab and resuspend it in 200 µL of buffer.

5. Serially dilute the cell resuspension in TSB with multiples of 10.

6. Plate the serially diluted cell suspensions and incubate them for overnight at 37^o^ C.

7. Count the colonies after overnight incubation and calculate the cell number on the respective spot on the plate at the time of harvesting.

5. After their respective incubation times, repeat steps 4 to 7 for P#1, P#2, P#3, and P#4.

6. Determine the growth rate by calculating the slope of the number of cells in P#0, P#1, P#2, P#3, and P#4 versus time plot on a Log_2_-linear plot.

The number of cells harvested from a plate, inoculated with 1 μL of nominally 10^5^ CFU/mL of cell stock, after incubation by a selected time in 0 to 6 h interval is presented in Fig A. As the vertical axis is the Log_2_ of the harvested cell number, each unit corresponds to a cell division cycle and the slope of the trendline, 2.8, indicates the number of division cycles per every hour of incubation. This particular knowledge is important when attempting to relate the actual cell number (biomass) to more easily measurable characteristics of colonies, such as average size, for monitoring colony growth dynamics.


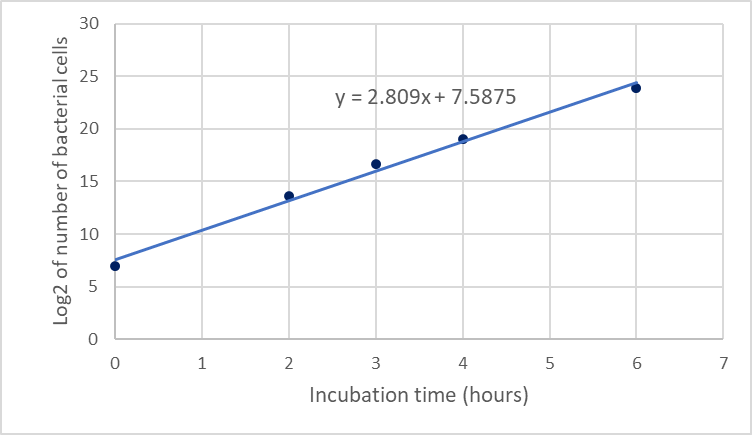


**Fig A. The number of microbial cells grown on a blood agar plate after dispensing 1 μL of nominally 10^5^ CFU/mL of cell stock on the plate and incubating in 37 ^o^C.** The slope of the trendline indicates the number of cell division cycles, and the y-intercept at ~7 corresponds to the number of cells (~120) dispensed on the plate.
