# Supplementary material for: Growth dynamics of Escherichia coli cells on a surface having AgNbO3 antimicrobial particles
Source: PLoS One. 2024 Aug 19;19(8):e0305315. doi: 10.1371/journal.pone.0305315 (PMC11332949; doi:10.1371/journal.pone.0305315)
Supplement: S2 Appendix — (DOCX) [file pone.0305315.s002.docx]

# **S2 Appendix. Surface density of AgNbO_3_ particles on antimicrobial gels**

The number density of the antimicrobial particles at the surface of the gel were verified by direct counting. For each of the two images of each gel containing AgNbO_3_ particles, two circular areas of diameter 100 μm were selected. The number of discernable particles within the areas were counted and the average of the four counts were calculated. The average particle count of gels containing 5 ng/mm^2^, 10 ng/mm^2^ and 20 ng/mm^2^ of AgNbO_3_ particles was respectively 38, 81 and 152. The particle count in a given area may be used to estimate the surface particle density of the two gel conditions according to the equation:

$\rho_{surface particle}= \frac{Particle count within dashed circle}{Area of dashed circle}$ (1)

Accordingly;

$$\rho_{surface particle \left( 5 ng/{mm}^{2} gel \right) = \frac{38}{7850 {\mu m}^{2}} \times\frac{1000000 {\mu m}^{2}}{1 {mm}^{2}} \sim4.8 \times{10}^{3} particles/{mm}^{2}}$$

$$\rho_{surface particle \left( 10 ng/{mm}^{2} gel \right) = \frac{81}{7850 {\mu m}^{2}} \times\frac{1000000 {\mu m}^{2}}{1 {mm}^{2}} \sim1.0 \times{10}^{4} particles/{mm}^{2}}$$

$$\rho_{surface particle \left( 20 ng/{mm}^{2} gel \right) = \frac{152}{7850 {\mu m}^{2}} \times\frac{1000000 {\mu m}^{2}}{1 {mm}^{2}} \sim1.93 \times{10}^{4} particles/{mm}^{2}}$$

The method above was verified by performing the following ImageJ operation on the rectangular section of the gel surface having 5 ng/mm^2^ of AgNbO_3_ particles as presented on the left side of Fig A:

1. Convert image type to 8-bit.
2. Select color and invert LUT.
3. Adjust threshold such that the majority of visible particles on the original image become. visible in the new image without merging. This is shown on the right side of Fig A.
4. Select “analyze” and analyze particles.


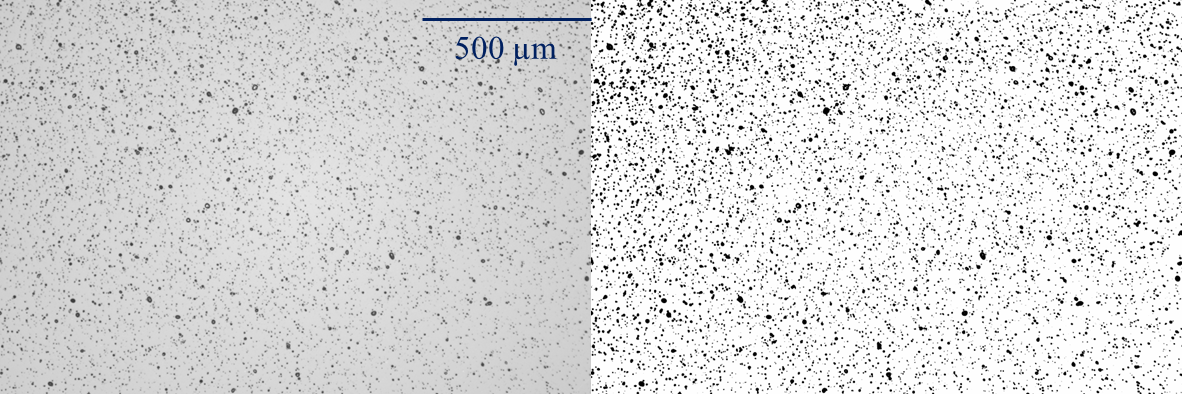


**Fig A. Left) The image of a 1.75mm × 1.17mm rectangular section of the gel surface having 5 ng/mm^2^ of AgNbO_3_ particles. Right) The image after applying transformations aimed at determining particle distribution.**

Employing this procedure, the particle count was found to be 10037 for the 5 ng/mm^2^ gel, corresponding to a particle density of ~ 4.9 × 10^3^ particles/mm^2^, close to the number acquired by direct counting.
