# Supplementary material for: Growth dynamics of Escherichia coli cells on a surface having AgNbO3 antimicrobial particles
Source: PLoS One. 2024 Aug 19;19(8):e0305315. doi: 10.1371/journal.pone.0305315 (PMC11332949; doi:10.1371/journal.pone.0305315)
Supplement: S3 Appendix — (DOCX) [file pone.0305315.s003.docx]

# **S3 Appendix. Estimating average particle size, volume and mass**

Using the obtained number density of particles at the gel surface from equation 1 of S2 Appendix, back calculation may be performed to estimate the average particle diameter at the surface of the gel:

$N= \frac{M_{d}}{\rho V}= \frac{M_{d}}{\rho\frac{4}{3}\pi r^{3}}$ (1)

$r= \sqrt[3]{\frac{3}{4}(\frac{M_{d}}{\pi\rho N})}$ (2)

$d=2r$ (3)

Where N, M_d_, ρ, V, r, d respectively represent surface particle density, mass of particles dispensed on gel per mm^2^, average particle mass density, average particle volume, average particle radius, and average particle diameter. The average recorded mass density of the AgNbO_3_ particles measured using a helium pycnometer was 6.02 ± 0.1 g/cm^3^, close to the reported mass density of 6.8 g/cm^3^ for AgNbO_3_ particles without ball milling treatment [1]. Therefore, taking 4.8 × 10^3^ particles/mm^2^ as the surface particle density corresponding to the 5 ng/mm^2^ gel, obtained from equation 1 of S2 Appendix, we obtain the particle diameter:

$d=2 \times\sqrt[3]{\frac{3}{4}\left( \frac{5.0 \times{10}^{-15}\frac{g}{\mu m^{2}}}{\left( 3.14 \right)\left( 6.02 \times{10}^{-12}\frac{g}{\mu m^{3}} \right)\left( 4.8 \times{10}^{-3} \frac{1}{\mu m^{2}} \right)} \right)} \sim0.69 \mu m$

This is indeed sufficiently close to the value of 0.44 μm we measured employing Dynamic Light Scattering (DLS) [2], considering the tendency for particle agglomeration over the gel surface.

Using the estimated average diameter, the average volume of the of the AgNbO_3_ particles can then be calculated according to the equation:

$V= \frac{4}{3}\pi{(\frac{d}{2})}^{3}$ (4)

Therefore;

$V= \frac{4}{3}\pi{(\frac{0.69}{2})}^{3} \sim0.17 {\mu m}^{3}$

Knowing the recorded mass density of the AgNbO_3_ particles, and the calculated average volume, the average mass of each AgNbO_3_ particles (M) can be obtained according to the equation:

$M= \rho\times V$ (5)

Therefore;

$M=\left( 6.02 \times{10}^{-12}\frac{g}{{\mu m}^{3}} \right)\times\left( 0.17 {\mu m}^{3} \right)=1.0 \times{10}^{-12} g=1.0 \times{10}^{-3}ng$

# **References**

1. Tian Y, Jin L, Zhang H, Xu Z, Wei X, Politova ED, et al. High energy density in silver niobate ceramics. Journal of Materials Chemistry A. 2016; 4(44), 17279-17287. doi: 10.1039/C6TA06353E

2.  Talebpour C, Fani F, Ouellette M, Salimnia H, Alamdari H. Nondegradable Antimicrobial Silver-Based Perovskite. ACS Sustainable Chem. Eng. 2022; 10 (15), 4922-4928. doi: 10.1021/acssuschemeng.1c08181
