# Supplementary material for: Growth dynamics of Escherichia coli cells on a surface having AgNbO3 antimicrobial particles
Source: PLoS One. 2024 Aug 19;19(8):e0305315. doi: 10.1371/journal.pone.0305315 (PMC11332949; doi:10.1371/journal.pone.0305315)
Supplement: S4 Appendix — (DOCX) [file pone.0305315.s004.docx]

# **S4 Appendix. Determining the equivalent MIC**

As presented in section 3.3 of the manuscript, the gel surface with AgNbO_3_ loading of 20 ng/mm^2^ was determined as the surface MIC. This gel loading corresponds to surface particle density of ~1.93 × 10^4^ particles in a given area of 1 mm^2^, as determined in S2 Appendix. We may use this number to calculate the volume particle density in a given volume of 1 mm^3^ according to the equation:

$\rho_{volume particle}= {(\rho_{surface particle})}^{\frac{3}{2}}$ (1)

The number of particles at the bulk of a 1 mm^3^ gel required for MIC is therefore:

$\rho_{volume particle}(20 ng/{mm}^{2} gel)={(1.93 \times{10}^{4} particles/{mm}^{2})}^{\frac{3}{2}} \sim2.68 \times{10}^{6} particles/{mm}^{2}$

Multiplying the volume particle density by the average mass of the particles returns the equivalent volume MIC:

$$Equivalent MIC\left( 20 ng/mm2 gel \right)=\rho_{volume particle}\left( 20 ng/mm2 gel \right)\times M$$

$=(2.68 \times{10}^{6} \frac{1}{{mm}^{3}})\times(1.0 \times{10}^{-3} ng)$

$\sim2.7 \times{10}^{3} ng/{mm}^{3}$

$\sim2.7 mg/mL$
