# Supplementary material for: Growth dynamics of Escherichia coli cells on a surface having AgNbO3 antimicrobial particles
Source: PLoS One. 2024 Aug 19;19(8):e0305315. doi: 10.1371/journal.pone.0305315 (PMC11332949; doi:10.1371/journal.pone.0305315)
Supplement: S5 Appendix — (DOCX) [file pone.0305315.s005.docx]

# **S5 Appendix. Determining distance between particles**

As supplementary data, the average distances between the particles were calculated by the formula for linear density:

$Distance between particles= \frac{1}{\sqrt{\rho_{surface particle}}}$ (1)

Accordingly,

$Distance between particle (5 gn/mm2 gel)= \frac{1}{\sqrt{4.8 \times{10}^{3} {mm}^{2}}} \times\frac{1000 \mu m}{1 mm}=14 \mu m$

$Distance between particle (10 gn/mm2 gel)= \frac{1}{\sqrt{1.0 \times{10}^{4} {mm}^{2}}} \times\frac{1000 \mu m}{1 mm}=9.8 \mu m$

$Distance between particle (20 gn/mm2 gel)= \frac{1}{\sqrt{1.93 \times{10}^{3} {mm}^{2}}} \times\frac{1000 \mu m}{1 mm}=7.20 \mu m$

According to our model, the average distance between particles should be 7.20 µm or less to achieve MIC.
