# Supplementary material for: Growth dynamics of Escherichia coli cells on a surface having AgNbO3 antimicrobial particles
Source: PLoS One. 2024 Aug 19;19(8):e0305315. doi: 10.1371/journal.pone.0305315 (PMC11332949; doi:10.1371/journal.pone.0305315)
Supplement: S6 Appendix — (DOCX) [file pone.0305315.s006.docx]

# **S6 Appendix. Supplementary information for Fig 6**

The exemplary microscopic images of a rectangular section on the gel, after incubation for 5 h, are presented in Figs A, B, C, and D, respectively for control, 5 ng/mm^2^, 10 ng/mm^2^, and 20 ng/mm^2^ gels. The colonies, whose detailed time-laped contours were shown in Fig 6, are indicated by numbers.


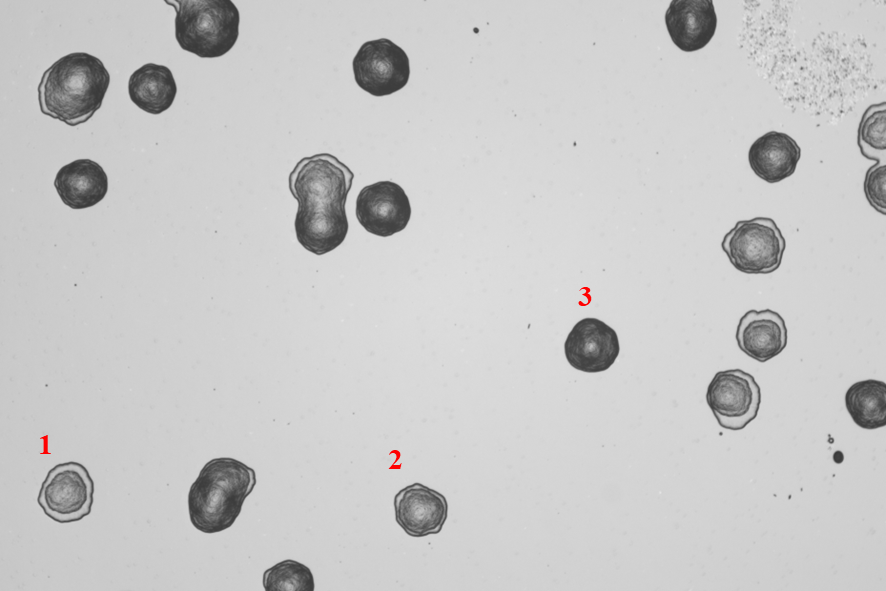


**Fig A. The image of a 1.75 mm × 1.17 mm rectangular section of the gel with no AgNbO_3_ particles, after 5 h of incubation.**


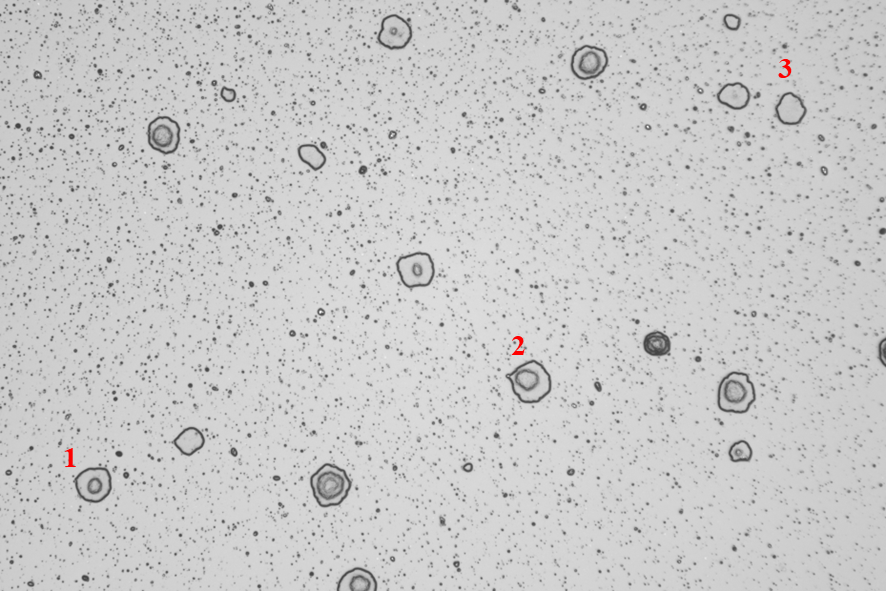


**Fig B. The image of a 1.75 mm × 1.17 mm rectangular section of the gel with 5 ng/mm^2^ of AgNbO_3_ particles, after 5 h of incubation.**


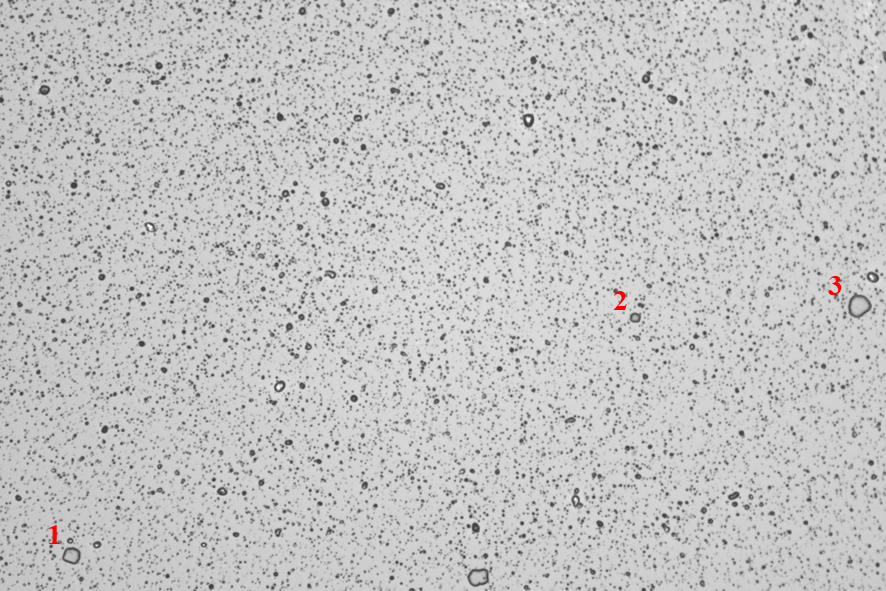


**Fig C. The image of a 1.75 mm × 1.17 mm rectangular section of the gel with 10 ng/mm^2^ of AgNbO_3_ particles, after 5 h of incubation.**


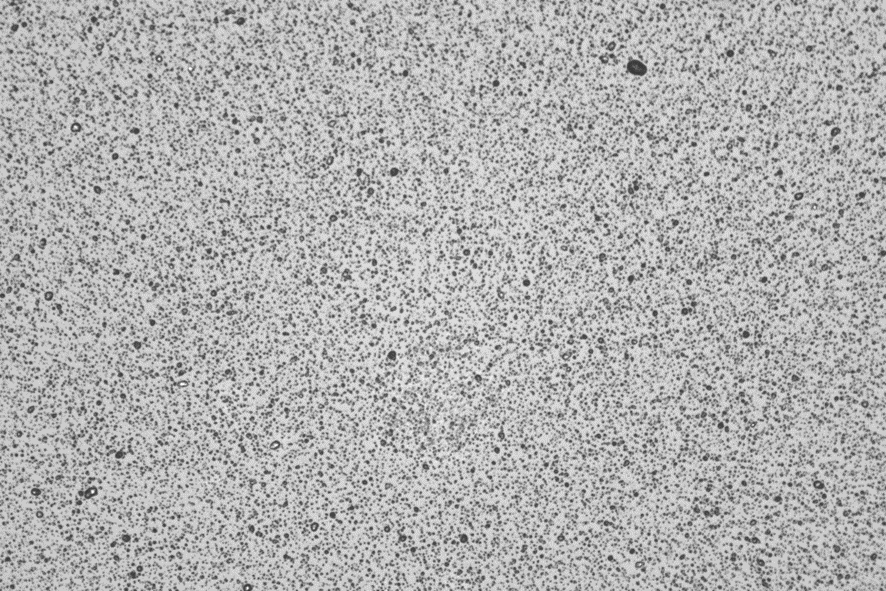


**Fig D. The image of a 1.75 mm × 1.17 mm rectangular section of the gel with 20 ng/mm^2^ of AgNbO_3_ particles, after 5 h of incubation.**
