# Supplementary material for: Growth dynamics of Escherichia coli cells on a surface having AgNbO3 antimicrobial particles
Source: PLoS One. 2024 Aug 19;19(8):e0305315. doi: 10.1371/journal.pone.0305315 (PMC11332949; doi:10.1371/journal.pone.0305315)
Supplement: S7 Appendix — (DOCX) [file pone.0305315.s007.docx]

**S7 Appendix. More data on the evolution of colonies**

In the discussion section, we stated that at particle concentration below MIC, the seeded cells, after a lag time of order 1-2 h, proceed with proliferation as if no particle were present afterwards. In this section, we further strengthen this statement by presenting the picture of 12 colonies at t = 5 h after incubation overlaid with the counters of the colonies at two earlier times of 2 and 4 h. The goal is to qualitatively illustrate that the presence of a large particle aggregate, presented in the pictures by the overlaid circles, doesn’t slow down the rate at which the surface area (biomass) of the colony increases. To this end, we selected an image similar to the ones presented in S6 Appendix but taken from the replica experiment. The image, which is presented in Fig A, includes 18 colonies. We excluded 5 colonies for the reason of being located in the edges or too much overlap with its neighbor and selected the remaining 12 colonies for qualitatively interrogating their temporal evolution. Corresponding to each colony we prepared an evolution image (see Fig B) following the procedure described below:

1. Draw the counter on the image taken after 2 h of incubation and fill it with violet color.
2. Locate those particles or their aggregates which are located in the vicinity of the colony and represent them with a circle having approximately similar size.
3. Remove the actual image and leave behind the colony contour and the circles representing the particles or their aggregates.
4. Overlay the shapes from step 3 on the image taken after 4 h of incubation.
5. Draw the colony counter (red line) and remove the actual image and leave behind the shapes.
6. Overlay the shapes from step 5 on the image taken after 5 h of incubation.
7. Overlay circles (dashed circles in Fig B) to ease judging the actual size of the colony at the three time points (2, 4, and 5 h)


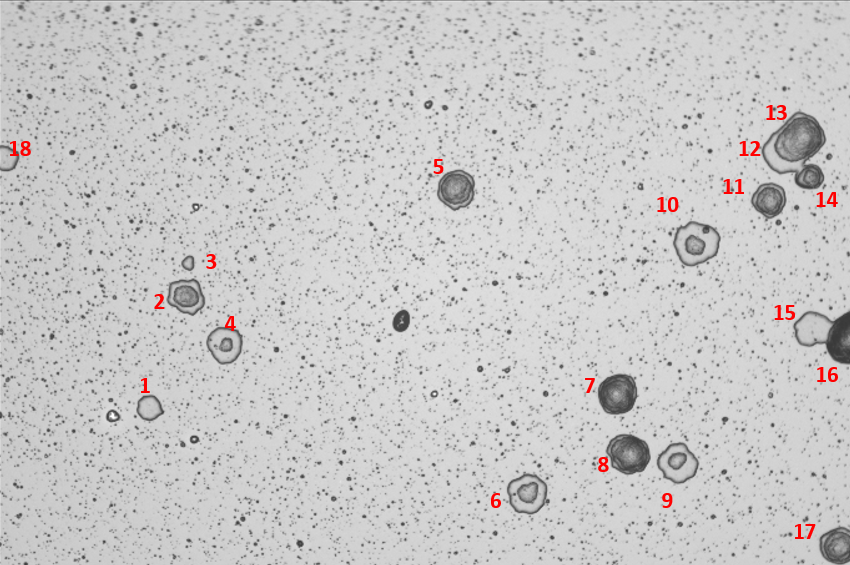


**Fig A. The image of a 1.75 mm × 1.17 mm rectangular section of the gel with 5 ng/mm^2^ of AgNbO_3_ particles, after 5 h of incubation**. The colonies with images shown in Fig B are labeled in accordance with the numbers on this picture.


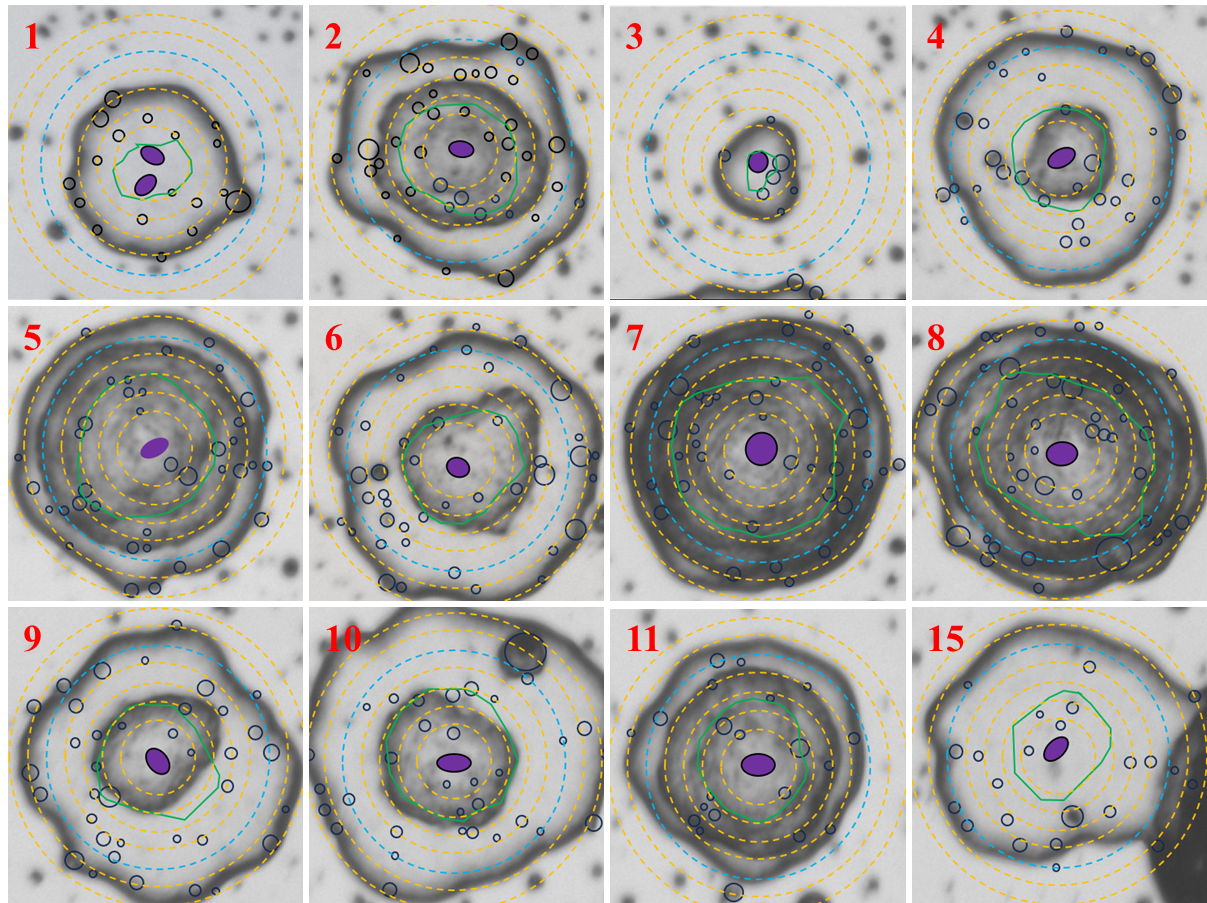


**Fig B. The photos of 15 colonies after 5 h of incubation on a spot with particle concentration of 5 ng/mm^2^.** The photos are overlaid with particle aggregates represented by proportionally sized circles. Also represented is the contour of the colony at earlier incubation times (violet 2 h, green 4 h). The dashed scale circles start from d = 10 μm for the innermost and continue and continue with 10 μm increment ending with d = 70 μm for the outermost. The diameter of the dashed blue circle is 50 μm.
