# Supplementary material for: Growth dynamics of Escherichia coli cells on a surface having AgNbO3 antimicrobial particles
Source: PLoS One. 2024 Aug 19;19(8):e0305315. doi: 10.1371/journal.pone.0305315 (PMC11332949; doi:10.1371/journal.pone.0305315)
Supplement: S8 Appendix — (DOCX) [file pone.0305315.s008.docx]

# **S8 Appendix. Size distribution and density of the antimicrobial particles on the bone cement**

To obtain a smooth surface for electron microscopy, the axial region of the PMMA - 1% AgNbO_3_ disk was subjected to sequential wet polishing with silicon carbide papers of grid number 240, 320, 400, 600, 800, followed by diamond suspension (Leco Corp) of 6 then 1 μm. For examination by Scanning Electron Microscopy (SEM), the polished samples were sputter-coated with an approximately 25 nm layer of graphite to enable electron conductivity. SEM images were obtained using a Vega 3 (Tescan) scanning electron microscope. Backscatter electron (BSE) imaging mode was opted as the imaging approach given its sensitivity to differences in atomic weight between materials of a composite surface. Imaging was performed with a potential of 15 kV and magnification levels of 500×, 1500×. As seen in Fig A, images in BSE return heavy elements as white speckles and a gray background consisting of lighter elements. To verify that the white speckles are indeed AgNbO_3_ particles, spot-size of Energy Dispersive Spectroscopy (EDS) at the location white speckles was performed with an EDAX Element EDS Detector, at an X-ray energy detection range of 0-7 keV, a resolution of 128.8 eV, and a magnification of 1500×.


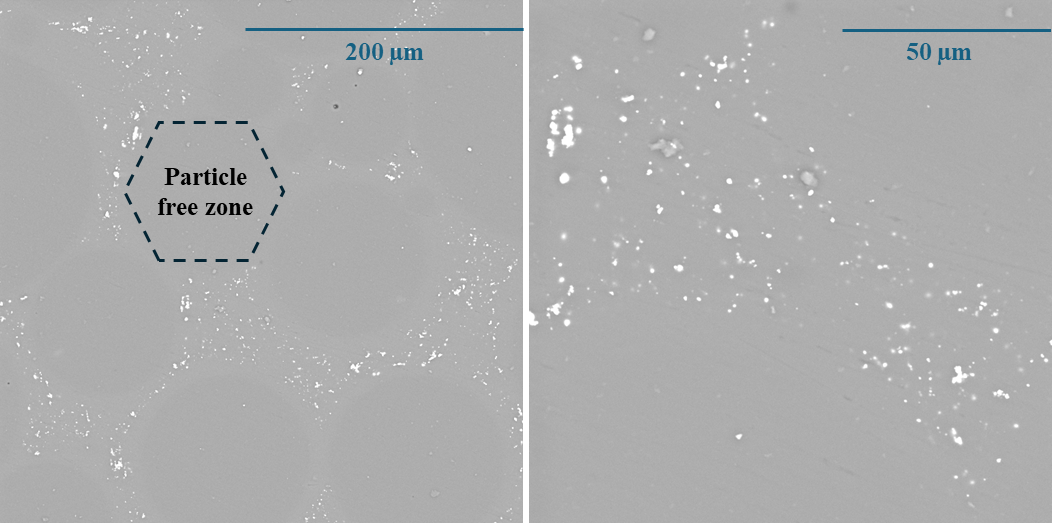


**Fig A. The SEM image of the polished axial surface of the PMMA - 1% AgNbO_3_ disk.** (Left to right): Magnification of 500×, 1500×.

A significant feature observed in Fig A is markedly inhomogeneous distribution of AgNbO_3_ particles (white spots), characterized by zones where there are no particles. In contrast, the particles are distributed over areas between these particle-free zones. This results from the manner by which the bone cement forms from its raw materials, including a liquid component and a dry component.

The liquid component of the bone cement consists of the liquid monomer of methyl methacrylate (MMA) and an accelerator, such as an amine (e.g., N,N-dimethyl-p-toluidine). The dry component of the bone cement includes an acrylic polymer, such as polymethyl methacrylate (PMMA) in the form of small polymer beads and a polymerization initiator, such as benzoyl peroxide, to trigger polymerization when mixing occurs. When the liquid component is mixed with the dry component, the liquid monomer wets the polymeric powder and the partial dissolution and swelling of the solid polymer beads in the liquid monomer occurs and shortly afterwards the hardening phase starts. Obviously, the bead content of the dry component only partially dissolves and the remaining part of the beads end up as a region devoid of particles. In addition, due to hydrophilic interaction the particles are uniformly distributed in inter-bead regions. The polishing procedure performed for preparing the PMMA surface for electron microscopy exaggerates the contribution of particle-free zones. Apparently, on an unpolished and rough surface particles will appear more or less uniformly distributed over the surface.

The size and distribution of the particles were performed according to the following ImageJ operation on the 1500× BSE image:

1. Convert image type to 8-bit.
2. Select color and invert LUT.
3. Adjust threshold such that the majority of visible particles on the original image become. visible in the new image without merging.
4. Select color and invert LUT
5. Select “analyze” and analyze particles.

Employing this procedure, the particle count was found to be 404, corresponding to a particle density of ~25000 particles/mm^2^, and an average particle size of 0.88 µm, close but slightly higher than the calculated average particle diameter of 0.69 µm of supplementary section 3. This enlargement can be attributed to occasional particle aggregation during the polymerization process.
