# Supplementary material for: Growth dynamics of Escherichia coli cells on a surface having AgNbO3 antimicrobial particles
Source: PLoS One. 2024 Aug 19;19(8):e0305315. doi: 10.1371/journal.pone.0305315 (PMC11332949; doi:10.1371/journal.pone.0305315)
Supplement: Attachment — Submitted filename: PONE-D-24-21073.pdf-With comments.pdf [file pone.0305315.s009.pdf]

PLOS ONE

Bacterial growth dynamics on a surface having a particulate antimicrobial agent

--Manuscript Draft--

**Manuscript Number:**	PONE-D-24-21073
**Article Type:**	Research Article
**Full Title:**	Bacterial growth dynamics on a surface having a particulate antimicrobial agent
**Short Title:**	Particulate antimicrobial agent
**Corresponding Author:**	Cyrus Talebpour, M.Sc.<br>Université Laval Faculté des Sciences et de Génie: Universite Laval Faculte des sciences et de genie<br>Québec, Quebec CANADA
**Keywords:**	AgNbO3; Antimicrobial; surface; Colony morphology; PMMA; Bone cement
**Abstract:**	The morphological dynamics of microbial cell proliferation on an antimicrobial surface at an early growth stage was studied with Escherichia coli on the surface of a gel supplied with nanostructured AgNbO3 antimicrobial particles. We demonstrated an inhibitory surface concentration, analogous to minimum inhibitory concentration, beyond which the growth of colonies and formation of biofilm are inhibited. In contrast, at lower concentrations, colonies circumvent the antimicrobial activity of the particles and grow with a short lag time of a few hours. Data obtained from the experiments on the gel surface were used to determine the concentration of the antibacterial particles in polymethyl methacrylate PMMA bone cement.
**Order of Authors:**	Cyrus Talebpour, M.Sc.
	Fereshteh Fani, PhD
	Hossein Salimnia, PhD
	Marc Ouellette, PhD
	Houshang Alamdari, PhD
**Additional Information:**	
**Question**	**Response**
**Financial Disclosure**<br><br>Enter a financial disclosure statement that describes the sources of funding for the work included in this submission. Review the submission guidelines for detailed requirements. View published research articles from *PLOS ONE* for specific examples.<br><br>This statement is required for submission and **will appear in the published article** if the submission is accepted. Please make sure it is accurate.	Yes

Powered by Editorial Manager® and ProduXion Manager® from Aries Systems Corporation

**Funded studies**<br>Enter a statement with the following details:<br>• Initials of the authors who received each award<br>• Grant numbers awarded to each author<br>• The full name of each funder<br>• URL of each funder website<br>• Did the sponsors or funders play any role in the study design, data collection and analysis, decision to publish, or preparation of the manuscript?<br><br>Did you receive funding for this work?	
Please add funding details.<br>as follow-up to "**Financial Disclosure**<br><br>Enter a financial disclosure statement that describes the sources of funding for the work included in this submission. Review the submission guidelines for detailed requirements. View published research articles from *PLOS ONE* for specific examples.<br><br>This statement is required for submission and **will appear in the published article** if the submission is accepted. Please make sure it is accurate.<br><br>**Funded studies**<br>Enter a statement with the following details:<br>• Initials of the authors who received each award<br>• Grant numbers awarded to each author<br>• The full name of each funder<br>• URL of each funder website<br>• Did the sponsors or funders play any role in the study design, data collection and analysis, decision to publish, or preparation of the manuscript?<br><br>Did you receive funding for this work?"	This study was financed by a Natural Sciences and Engineering Research Council of Canada (https://www.nserc-crsng.gc.ca/index_eng.asp) grant No. RGPIN-2023-04795 to H.A. and by the Canadian Institutes of Health Research (https://cihr-irsc.gc.ca/e/193.html) foundation grant No. FND167283 to M.O.. M.O. is the holder of a<br>Canada Research Chair in Antimicrobial Resistance. The sponsors did not play any role in the study design, data collection and analysis, decision to publish, or preparation of the manuscript.
Please select the country of your main research funder (please select carefully as in some cases this is used in fee calculation).	CANADA - CA

Powered by Editorial Manager® and ProduXion Manager® from Aries Systems Corporation

as follow-up to "**Financial Disclosure**<br><br>Enter a financial disclosure statement that describes the sources of funding for the work included in this submission. Review the submission guidelines for detailed requirements. View published research articles from *PLOS ONE* for specific examples.<br><br>This statement is required for submission and **will appear in the published article** if the submission is accepted. Please make sure it is accurate.<br><br>**Funded studies**<br>Enter a statement with the following details:<br>• Initials of the authors who received each award<br>• Grant numbers awarded to each author<br>• The full name of each funder<br>• URL of each funder website<br>• Did the sponsors or funders play any role in the study design, data collection and analysis, decision to publish, or preparation of the manuscript?<br><br>Did you receive funding for this work?"	
**Competing Interests**<br><br>Use the instructions below to enter a competing interest statement for this submission. On behalf of all authors, disclose any competing interests that could be perceived to bias this work—acknowledging all financial support and any other relevant financial or non-financial competing interests.<br><br>This statement is required for submission and **will appear in the published article** if the submission is accepted. Please make sure it is accurate and that any funding sources listed in your Funding Information later in the submission form are also declared in your Financial Disclosure statement.	The authors have declared that no competing interests exist.

View published research articles from *PLOS ONE* for specific examples.<br><br>**NO authors have competing interests**<br><br>Enter: *The authors have declared that no competing interests exist.*<br><br>**Authors with competing interests**<br><br>Enter competing interest details beginning with this statement:<br><br>*I have read the journal's policy and the authors of this manuscript have the following competing interests: [insert competing interests here]*<br><br>* typeset	
**Ethics Statement**<br><br>Enter an ethics statement for this submission. This statement is required if the study involved:<br><br>• Human participants<br>• Human specimens or tissue<br>• Vertebrate animals or cephalopods<br>• Vertebrate embryos or tissues<br>• Field research<br><br>Write "N/A" if the submission does not require an ethics statement.<br><br>General guidance is provided below. Consult the submission guidelines for detailed instructions. **Make sure that all information entered here is included in the Methods section of the manuscript.**	N/A

**Format for specific study types**<br><br>**Human Subject Research (involving human participants and/or tissue)**<br>• Give the name of the institutional review board or ethics committee that approved the study<br>• Include the approval number and/or a statement indicating approval of this research<br>• Indicate the form of consent obtained (written/oral) or the reason that consent was not obtained (e.g. the data were analyzed anonymously)<br><br>**Animal Research (involving vertebrate animals, embryos or tissues)**<br>• Provide the name of the Institutional Animal Care and Use Committee (IACUC) or other relevant ethics board that reviewed the study protocol, and indicate whether they approved this research or granted a formal waiver of ethical approval<br>• Include an approval number if one was obtained<br>• If the study involved *non-human primates*, add *additional details* about animal welfare and steps taken to ameliorate suffering<br>• If anesthesia, euthanasia, or any kind of animal sacrifice is part of the study, include briefly which substances and/or methods were applied<br><br>**Field Research**<br><br>Include the following details if this study involves the collection of plant, animal, or other materials from a natural setting:<br>• Field permit number<br>• Name of the institution or relevant body that granted permission	
**Data Availability**<br><br>Authors are required to make all data underlying the findings described fully available, without restriction, and from the time of publication. PLOS allows rare exceptions to address legal and ethical concerns. See the PLOS Data Policy and FAQ for detailed information.	Yes - all data are fully available without restriction

Powered by Editorial Manager® and ProduXion Manager® from Aries Systems Corporation

<table>
<tr><td>A Data Availability Statement describing where the data can be found is required at submission. Your answers to this question constitute the Data Availability Statement and will be published in the article, if accepted.

Important: Stating ‘data available on request from the author’ is not sufficient. If your data are only available upon request, select ‘No’ for the first question and explain your exceptional situation in the text box.

Do the authors confirm that all data underlying the findings described in their manuscript are fully available without restriction?</td><td></td></tr>
<tr><td>Describe where the data may be found in full sentences. If you are copying our sample text, replace any instances of XXX with the appropriate details.

• If the data are held or will be held in a public repository, include URLs, accession numbers or DOIs. If this information will only be available after acceptance, indicate this by ticking the box below. For example: All XXX files are available from the XXX database (accession number(s) XXX, XXX.).
• If the data are all contained within the manuscript and/or Supporting Information files, enter the following: All relevant data are within the manuscript and its Supporting Information files.
• If neither of these applies but you are able to provide details of access elsewhere, with or without limitations, please do so. For example:

Data cannot be shared publicly because of [XXX]. Data are available from the XXX Institutional Data Access / Ethics Committee (contact via XXX) for researchers who meet the criteria for access to confidential data.

The data underlying the results presented in the study are available from (include the name of the third party</td><td>All relevant data are within the manuscript and its Supporting Information files.</td></tr>
</table>

and contact information or URL).<br>• This text is appropriate if the data are owned by a third party and authors do not have permission to share the data.<br><br>* typeset	
Additional data availability information:	

Manuscript Click here to access/download;Manuscript;Manuscript.docx

Bacterial growth dynamics on a surface having a particulate antimicrobial agent

Cyrus Talebpour[1¶*], Fereshteh Fani[2¶], Hossein Salimnia[3¶], Marc Ouellette[2¶], Houshang Alamdari[1¶]

[1] Department of Mining, Metallurgical and Materials Engineering, Université Laval Faculté des Sciences et de Génie, Québec, Quebec, Canada

[2] Department of Microbiology and Immunology, Université Laval Faculté de Médecine, Québec, Quebec, Canada

[3] Department of Pathology, Children's Hospital of Michigan, Detroit, Michigan, United States of America

*Corresponding author

E-mail: cyrus.talebpour.1@ulaval.ca (CT)

[¶]These authors contributed equally to this work.

Abstract

The morphological dynamics of microbial cell proliferation on an antimicrobial surface at an early growth stage was studied with *Escherichia coli* on the surface of a gel supplied with nanostructured $AgNbO_3$ antimicrobial particles. We demonstrated an inhibitory surface concentration, analogous to minimum inhibitory concentration, beyond which the growth of colonies and formation of biofilm are inhibited. In contrast, at lower concentrations, colonies circumvent the antimicrobial activity of the particles and grow with a short lag time of a few hours. The applicability of these findings, in terms of estimating inhibitory surface concentration, was tested in the case of antimicrobial polymethyl methacrylate (PMMA) bone cement.

Keywords: $AgNbO_3$, Antimicrobial, surface, colony morphology, PMMA, bone cement

1. Introduction

The misuse of antibiotics has led to development of infections attributed to antimicrobial resistant (AMR) bacteria strains, which have been responsible for an annual 1.3 million deaths globally [1]. If nothing is done, this figure is projected to rise to 10 million by 2050 [2], with expenditures resulting in a global loss of $108 trillion USD [3]. The need to counter AMR may be achieved through strategies such as effectively reducing the microbial load on surfaces that come into contact with pathogenic microbial cells [4]. Any remedy for rendering antimicrobial properties to these surfaces is desired to present no systemic or localized toxicity, inhibit the proliferation of most species of microbial cells in contrast to a selected category of species, provide a robust system enabling ease-of-use, and involve a low production cost [5]. In this respect, silver coatings have been good candidates [6-8], thanks to their broad-spectrum antimicrobial activity through

mechanisms such as production of reactive oxygen species (ROS) [9]. However, this approach is associated with drawbacks such as incompatibility of the conventional coating techniques with some important solids, altering the structure of surface, and corrosion with elution of silver ions, which ironically are the agent of antimicrobial activity [10].

Recently, we found a possible solution for the latter drawback by synthesizing nano-structured $AgNbO_3$ nanoparticles, which, despite having diminished ion release rate, show strong antimicrobial activity [11]. This kind of antimicrobial activity, which we had defined as 'through contact', are associated with particulate agents and can be incorporated into the surface provided that the required amount of the particles is not below a threshold. Thus, a main question to be addressed for designing an antimicrobial surface employing these particles is introducing and quantifying a property akin to the minimum inhibitory concentration (MIC).

Another question, relevant for the antimicrobial surfaces in niche applications such as the interfacial surfaces of implants, is the formation and evolution of biofilms, i.e., structured communities of sessile bacteria [12] encased in a self-produced matrix of extracellular polymeric substances (EPS) [13]. In the context of AMR, the biofilm structure facilitates quorum sensing between cells responsible for altering gene expression, accumulating genetic material for cellular uptake, promoting horizontal gene transfer, and enabling less metabolically active cells known as persister cells to arise [14]. Up to 65-80% of all infections are associated with biofilm formation, much of which are implicated in chronic infections, in contrast to planktonic bacteria involved in acute processes [15]. Therefore, a phenomenological investigation of biofilm evolution on a surface having a concentration of antimicrobial particles sublethal to bacteria is a minimum necessity for actual implementation of an antimicrobial surface.

In this study we determine the surface minimum inhibitory concentration (SMIC) for surface-utilizing particles with antimicrobial activity through contact. The utility of these findings is illustrated in the case of polymethyl methacrylate (PMMA) bone cement. Furthermore, a qualitative description of colony formation and expansion at early stages are presented by time lapse microscopy on a model system, consisting of a gel having dispersed $AgNbO_3$ nanoparticles on its surface. It is worth mentioning that incorporation of the right amount of $AgNbO_3$ nanoparticles in PMMA bone cement is expected to confer permanent antibacterial property to the cement due to its extremely limited corrosion/dissolution rate, contrary to the incorporation of conventional antibiotics such as gentamicin.

2. Methodology

2.1 Preparation and characterization of nanostructured antimicrobial particles

Nanostructured $AgNbO_3$ antimicrobial particles were synthesized using the Activated Reactive Synthesis (ARS) method as described previously [11, 16]. Briefly, the raw materials Ag_2O (Sigma-Aldrich Corp) and Nb_2O_5 (Inframat® Advanced Materials LLC), with the weight ratio of 1 g to 1.147 g respectively (for each 1 g of Ag_2O, 1.147 g of Nb_2O_5 powders), were mixed in a hardened steel crucible with high energy ball milling for 10 min. The mixture was transferred to a ceramic crucible and placed in an oven where it was gradually heated at a rate of 5 ^{o}C/min until the formation temperature, 1000 ^{o}C, was reached. The mixture was kept at this temperature for about 4 h and gradually cooled down at a rate of 10 ^{o}C/min to room temperature. The post-synthesis treatment included subjecting the powder to successive steps of high and low energy ball milling. The high energy ball milling was carried out using the 8000D Mixer/Mill® (SPEX SamplePrep,

LLC). An 8001 hardened steel crucible and a milling media consisting of two balls of 12.70 mm and one ball of 6.35 mm in diameter were used for milling 7 g of material in each batch at an oscillation rate of 1060 cycles/min for a duration of 90 min. Low energy ball milling was carried out by transferring approximately 40 g of powder from the previous step (agglomerates) to a crucible containing hundreds of steel beads of 4.5 mm in diameter, which were made to rotate at 90 rpm by Szegvari Attritor System Type E Model 01-STD (Union Process, Inc.). To this, 10 mL of water was added and the attrition process was performed for 120 min. At the end of the operation the beads were rinsed with deionized water and the residue thus obtained was dried inside an oven with a temperature of 150 ^{o}C overnight to obtain the final nanostructured $AgNbO_3$ particles. The particles were previously characterized in terms of size by the Malvern ZEN1600 Dynamic Light Scattering (DLS) machine and found to have an average size of 0.44 μm [11]. Density of the particles was measured using a AccuPyc II 1340 (Micromeritics Instruments Corp.) helium gas pycnometer. Powder samples of ~ 8 g were weighted in a 10 mL stainless steel cell, and the density was obtained by dividing the mass of the sample to the volume measured by the pycnometer.

2.2 Bacterial strain and culture conditions

The bacterial strain used in this study was *Escherichia coli* ATCC # 25922 and was prepared as described below. Cell stock in 50% glycerol was taken from a -80 ^{o}C freezer and after thawing, 30-50 µL of it was transferred to a Tryptic Soy Agar (TSA) (with 5% sheep blood) plate (the P1 plate), inoculated by standard streaking, and incubated at 37 ^{o}C overnight under aerobic condition until colonies were visible. A well-formed representative colony from the plate was picked and inoculated into 3 mL of Tryptic Soy Broth (TSB) and incubated at 37 ^{o}C with 150 rpm shaking for 3 h. Then, a 1 mL culture was transferred to 2 mL autoclaved tubes and centrifuged at 8000 rpm for 8 min in a microcentrifuge. The harvested cells were resuspended in 1 mL TSB and 0.5 mL

was used for OD_{600} measurement. Using an in-house OD_{600} vs cell count correlation database, a cell suspension of 10^8 CFU/mL was prepared. The suspension was then serially diluted in TSB to a final concentration of 10^7, 10^6 and 10^5 CFU/mL by pipetting and vortex mixing at each dilution step.

2.3 Planktonic growth curve and antimicrobial susceptibility

The growth of *Escherichia coli* ATCC # 25922 bacterial cells were determined using an automated incubation system (Biospa, BioTek) integrated with Cytation 5 multimode reader (BioTek). 10 µl of 1×10^7 CFU/mL bacteria cells were inoculated in 990 µl of LB medium in the absence or presence of $AgNbO_3$ nanoparticles at concentrations of 4, 8, 16 and 32 µg/ml in a Falcon® 24-well plate. The plate was incubated at 37°C, and the OD_{600} was read at each 1 h for 20 h after shaking for 10 s using a Cytation 5 multimode reader. Data was processed using Gen5 software and reported with GraphPad Prism. The minimum inhibitory concentration (MIC) was determined as the concentration of particles for which no growth was observed after 24 h of incubation. All MICs were determined from at least three independent biological replicates.

2.4 Preparation of antimicrobial gel surfaces

5 mg of the nanostructured particles was weighted and resuspended in 5 mL of deionized water. The suspension was serially diluted to concentrations of 200, 100, and 50 µg/mL. To prepare an antimicrobial gel, standard A9 blood agar plate was left on the table for 30 min, then 5 µL of each particle suspension, after vortexing for 1 min, was dispensed at a spot in the first half of the plate. The procedure was repeated on the second half of the plate to obtain two technical replicates. On each half of the plate, 5 µL of water was dispensed on designated spots as control. The dispensed particle suspension spreads to a disk with a diameter of ~ 8 mm and surface area of ~ 50 mm^2

before drying after 30 min. Thus, the particle density at the respective spots corresponding to 50 μg/mL, 100 μg/mL, and 200 μg/mL particle suspensions are respectively 5 ng/mm^2, 10 ng/mm^2, and 20 ng/mm^2. Three biological replicates were prepared for each condition.

2.5 Seeding microbial cells on solid phase growth media and interrogating their growth

From a 1 × 10^5 CFU/mL *Escherichia coli* ATCC # 25922 cell stock, 1 μL (nominally containing 100 CFU) was dispensed on each spot wherein particle suspensions and controls were previously dispensed. The dispensed cell stock spreads to a disk with a diameter of ~ 4 mm and surface area of ~ 12 mm^2. The plates were placed inside the incubator and a rectangular part at the center of the spots, having an area of 2.05 mm^2 (1.75 mm × 1.17 mm), were imaged with a metallurgical microscope (10× objective) in a bright and dark field at different time intervals. Each image represents approximately 1/6 of the entire spot area and the number of the growing colonies detected on it fairly well approximates 1/6 of the dispensed cells, verifying the homogenous dispersion of the bacteria cell on the surface. Using imageJ software, the microscopic images were analyzed for extracting the characteristic features of three representative colonies within one spot for each condition, and the average size of the 20 representative colonies from the two spots for each condition. Two other biological replicates for each condition were done for confirmation of the results obtained.

2.6 Preparing and testing antimicrobial disks

$AgNbO_3$ loaded polymethylmethacrylate (PMMA) disks were prepared by mixing 10 g of the PMMA dry component (A formulation of 97% Poly(methyl methacrylate), Sigma-Aldrich, and 3% Benzoyl peroxide, Sigma-Aldrich) with different amounts of $AgNbO_3$ (Control, 0.5%, 1%,

and 2%) to obtain the desired W/W ratio. Then, 5 ml of liquid monomer (A formulation of 97% Methyl methacrylate, Sigma-Aldrich, and 3% N,N-Dimethyl-p-toluidine, thermo scientific) was added to the mixture and mixed until a homogenous paste was formed. This was immediately cast in a High-Density Polyethylene (HDPE) mold with an inner diameter of 12 mm and depth of 10 mm. After solidification, the disc was released from the mold and used in subsequent experiments. The naming conventions for the samples are hereafter designated as control PMMA and PMMA - X% $AgNbO_3$ (X = 0.5, 1, 2).

The disks were tested for antibacterial properties following the CLSI M07 and CLSI M100 guidelines [17, 18]. Briefly, three replicates of each disk were sterilized by soaking in 70% isopropanol for one min, washing in sterile water for 2 min, and allowing them to air dry. The top surfaces of the disks were respectively loaded with a 50 µl of 1.5×10^6 CFU/mL *Escherichia coli* ATCC # 25922 bacterial cell suspensions (corresponding to around 75000 bacterial cells) and, after allowing to dry in air for 3 h, were dropped in a tube of 5 mL TSB. 5 min after dropping the disks in TSB, a 1 mL sample from each growth media was transferred in a Falcon ® 24-well plate. The original tubes were incubated at 37 ^{o}C overnight. After incubation, the tube was checked visually for signs of growth (turbidity) or lack of growth (clear medium). For the plate, the OD_{600} was read each 1 h for 20 h after shaking for 10 s using a Cytation 5 multimode reader (BioTek).

3. Results and discussion

3.1 Planktonic growth dynamics

Fig 1 presents the planktonic growth dynamics, indicated by average optical density over three biological replicates versus incubation time. Based on the general shape of the curves, the impact of antimicrobial particles on the cell viability, being manifested in the form of prolonging the lag

phase by 4 and 8 h, respectively for 4 and 8 μg/mL samples. This may be a manifestation of the "tolerance by lag" phenomenon, which appears to involve a generalized adaptive response to antibiotic stress [19]. Alternatively, one may attribute the prolonged lag phase to the presence of a subpopulation of antimicrobial persister cells in the entire inoculum 10^5 CFU that have a phenotypic tolerance to the antimicrobial particles [20]. The second key observation is that the minimum inhibitory value is at most 16 μg/mL, as there is no sign of growth (increase in OD_{600}) for this level of particle concentration after 20 h of incubation. Regarding these observations, some contrasts are expected with the case of exposing the microbial cells to antimicrobial particles on the surfaces for the following reasons. As the action of these particles is exerted via contact (since they do not release silver ions over the MIC level [11]) the growth inhibition on the surface has to occur either by incidental placing of a seeded cell at the proximity of a particle or via the cell motility leading to collisions.

Fig 1. The growth curve of *Escherichia coli* ATCC # 25922 in LB broth in the presence of antimicrobial $AgNbO_3$ particles with different concentrations. Each point is the average of three biological replicas.

3.2 Morphological and growth dynamics of colonies on a surface with no antimicrobial particles

The growth behavior of colonies on a plate with no antimicrobial particles performed as an independent test was selected as a baseline for assessing the impact of particles during the following experiments. The results are hereafter referred to as "trend". The parameters selected for this purpose were colony shape and its overall size, which is defined as the diameter, *d*, of a disk with similar area, as specified in equation 1.

$$\text{[illegible]} = 2\sqrt{\frac{\text{[illegible]}}{\text{[illegible]}}} \qquad (1)$$

When 1 µL of a 1×10^2 CFU/µL cell suspension was dispensed on the agar plate, 22 and 24 colonies were observed on the respective images taken from two spots. The area of the images of the two spots represented 1/6th of the total seeded area. The average of these two numbers, i.e., 23, indicates the number of colonies in the examined area. The total number of the colonies can then be estimated as $6 \times 23 = 138$, which is close to the nominally expected number of the dispensed cells. Fig 2 presents zoomed images of a typical selected colony taken at different times of incubation of (2-5) h. The contours of the colonies at different time intervals are superimposed and presented at the center of the figure. The images may be used to describe the phases of colony morphology. In the first 3 h of incubation, the colony has expanded into an irregular shape, which, based on dark field images (not presented here), is in the form of a monolayer sheet of cells. At t = 3 h, the area of the colonies, averaged over the 10 colonies across the images of the two spots, is 391 μm^2. After an extra hour of incubation, the average colony area increases to 2044 μm^2, which is an increase by a factor of 5.2 (~ 2.4 cycles). This factor is significantly smaller than the growth rate of 2.8 determined for microbial cells within a microcolony on an agar plate (See S1 Appendix). The deviation can be explained by noting that a second layer with a smaller area (presented by dashed green curve on the image corresponding to t = 4 h) was formed at the inner part of the first layer, thus increasing the total biomass of the colony by ~ 0.4 cycles. Further incubation time increases these inner layers and the incremental change in colony's apparent area drops to 3.6× (~ 1.9 cycles). This indicates a transition from the early stage of single layer colony to the stage when the colony is in the form of an incomplete cone with many layers [21, 22]. Based on the published research in the literature, the described trend in colony morphology results from

the bacterial motility [23-27] and formation of extracellular matrix and/or cellulose which might contain protein fibers [28].

Fig 2. The morphological dynamics of three colonies grown on the surface of an agar gel and monitored with time lapse microscopy over 5 h of incubation. The contours indicate the colony boundary in the following order: Blue oval in center: Colony at t = 1 h, Violet: 2 h, Yellow: 3 h, Green: 4 h, and Red: 5 h.

This qualitative observation suggests a phenomenological description of a colony as schematically presented in Fig 3. The colony starts with a rugged monolayer cell as suggested by a single curved bright boundary in the accompanying dark field image. Then, distinct cell layers are added on the top of the first layer (z-direction). As the colony grows further, more layers are added and their boundaries in the dark-field image lose distinction. According to this model, the growth on solid phase takes place in 3 stages: 1) Early development phase, during which the colony is in the form of a single layer cell sheet and does not yet exhibit clear characteristic features, 2) Intermediate development phase, during which distinct cell layers are formed over the first layer, and, 3) Mature phase, when the layered structure makes a transition to a rough mass of cells. Based on this view, during the early development phase the cells are in direct contact with the surface and if the antimicrobial activity of the surface is not sufficient to prevent cell proliferation in a time scale of less than a few growth cycles, the upper layers may form. If the antimicrobial activity is exerted by contact, one can anticipate that these top layers be protected from the antimicrobial surface via the first layer, which acts as a barrier.

Fig 3. The schematic layered structure of a typical colony over incubation time. Schematics adapted from the reference [29], along with recorded dark-field images of a typical colony.

3.3 Growth dynamics of colonies on a surface containing antimicrobial particles

Fig A-D of S6 Appendix presents the partial image of the inoculated spot for different surface concentrations of antimicrobial particles of (0, 5, 10 and 20) ng/mm^2 cases. The total number of colonies and the average size of 10 representative colonies grown on each of the two images for each condition after 6 h incubation were averaged and presented in Table 1. The images of the spots after leaving the plates at room temperature overnight, are presented in Fig 4.

Table 1. The average number and sizes of colonies grown on 1.75 mm × 1.17 mm rectangular sections of two inoculated spots and their size distribution after 6 h incubation.

Particle concentration (ng/mm^2)	# of colonies	Average size (µm)
**0**	21	150 ± 16
**5**	18	77 ± 19
**10**	4	48 ± 14
**20**	0	0

Fig 4. Shape of *Escherichia coli* colonies in two separate inocula on different BAP plates after leaving at room temperature overnight with varying concentrations of $AgNbO_3$.

The following observations were made: 1) 15% of the dispensed cells did not survive on the gel having 5 ng/mm^2 of $AgNbO_3$ particles. 2) The viability of the dispensed cells on the gel having 10 ng/mm^2 of particles was 19%, and 3) There is no sign of growth for 20 ng/mm^2. Accordingly, we took 20 ng/mm^2 as equivalent to the MIC in the case of planktonic growth represented by the curves in Fig 1, albeit this does not imply an equivalency as it is the case for

microdilution and agar dilution susceptibility tests for antibiotics [30]. The discrepancy results from the fact that the antimicrobial particles interact with bacterial cells not by diffusion but via contact.

Assuming an actual size of 0.69 μm for particles (See S3 Appendix), the surface MIC of 20 ng/mm^2 translates to ~ 2.7 mg/mL of $AgNbO_3$ (See S4 Appendix); a value much higher than the 16 μg/mL obtained for planktonic cells (Fig 1). As it will be later seen, this observation is in good agreement with the exemplary case of loading a composite solid with the antimicrobial particles. However, first report our observation with the growth behavior of bacteria on the surface of gels containing sub-MIC surface concentration of antimicrobial particles.

In Fig 5, the size of microcolonies (average and standard deviation over 10 representative colonies) are presented as a function of time and were compared to those measured for the trend obtained in an independent test, as described in the previous subsection. Three observations can be made from this figure: 1) As it is expected, the growth behavior of the bacteria on a gel, as indicated by average colony size, is well repeated (control vs trend), 2) the lag time increases with concentration of antimicrobial particles. However, the lag time is smaller on surfaces than that in planktonic cases. For example, at ¼ and ½ the surface MIC, i.e. gels with 5 ng/mm^2 and 10 ng/mm^2 nanoparticles, the lag phases are 1 and 2 h respectively (Fig 1). In the planktonic cases, at antimicrobial concentrations of ¼ and ½ the MIC, the lag phases are 4 and 8 h respectively. 3) Finally, and similarly to planktonic cells (Fig 1), the surviving cells on the surface proliferate with an exponential growth rate, similar to the case of no antimicrobial particles (Fig 5).

Fig 5. The average size of microbial cells grown on a gel surface having antimicrobial particles with surface densities of 5 and 10 ng/mm^2 as compared to the size of colonies grown on a normal gel.

In Fig 6, the cropped microscopic images and time-lapse contours at 2 h, 4 h, 5 h, and 6 h of three colonies for control, 5 ng/mm^2 and 10 ng/mm^2 gels are presented. A dashed circle representing a radius of 150 μm, 100 μm and 70 μm was overlaid on the time-lapse contours of colonies from the control, 5 ng/mm^2 and 10 ng/mm^2 of $AgNbO_3$ respectively. Within each of these dashed circles, the location of antimicrobial particles are presented as solid gray points. The original microscopic image of the gels after 5 h of incubation showing the location of the colonies are presented in Fig A-C in S6 Appendix.

Fig 6. Evolution of the morphology of three representative colonies in the presence of different antimicrobial particle concentrations. First row: no antimicrobial particles, Second row: having antimicrobial particles with a surface density of 5 ng/mm^2, and Third row: having antimicrobial particles with a surface density of 10 ng/mm^2. Color code: Violet (2 h), Green (4 h), Red (5 h), and Cyan (6 h). The images of the colony after different incubation times are also shown at the corners in each case (starting from 2 h at upper left corner moving counterclockwise: 2 h, 4 h, 5 h, and 6 h). The antimicrobial particles are represented by solid gray points.

In the case of the control gel, similar to the phenomenological description of colonies in the independent baseline test, the colony starts with a cell monolayer and its size logarithmically increases. After 4 h of incubation, more cell layers are added over the first layer and finally the colony matures by the formation of a rough biomass structure. For the case of growth on gels with antimicrobial particles, the general trend is similar to the control, except the events have been delayed by lag time. This implies that once the seeded cell is able to overcome the antimicrobial induced lag phase, it is able to proceed with proliferation as part of a biofilm.

We hypothesize that just after being seeded, the cell starts preparing for the exponential growth phase by trying to accumulate transient metals [31]. The presence of a silver-containing compound in the vicinity perturbs this process. However, if a cell could overcome this hurdle and

successfully divide, its descendants produce EPS, which acts as a structural barrier and provides tolerance to the antimicrobial particles [32]. In other words, the surface density of the particles must be sufficient enough to prevent the proliferation from the beginning, otherwise bacteria will create a protective barrier and continue to grow. This finding is of special importance in designing appropriate antibacterial surfaces, namely the bone cements which is our main goal. The bone cement must have sufficient particle density at its surface to prevent the proliferation of bacteria cells but not too high to be safe for human cells.

3.4 Bacterial proliferation on the surface of a composite solid loaded with antimicrobial particles

The predictive capability of our findings on the gel surface MIC was tested by assessing the antimicrobial level of a composite material. In this regard, we used PMMA as a solid, which is widely used as bone cement in orthopedic operations. The PMMA disks, loaded by different amounts of $AgNbO_3$ particles, were inoculated with ~ 75000 CFU of *Escherichia coli* and the survival of the cells were assessed. The result is summarized in Fig 7. The first observation is the 3 h of lag in the case of the PMMA - 0.5% $AgNbO_3$ bone cement. This is more similar to the ~ 2 h lag for the 10 ng/mm^2 gel (as indicated in Fig 5) than to the ~ 8 h of lag near MIC during the planktonic growth case of Fig 1. The second key observation is related to the surface MIC at the PMMA - 1% $AgNbO_3$. According to the data presented in S2 Appendix, the estimated surface density of the $AgNbO_3$ particles for 20 ng/mm^2 gel is 1.93×10^4 particles/mm^2. If the particles are loaded in the bulk gel, this would correspond to an equivalent volume of 2.68×10^{-3} g/mL loading (See S4 Appendix). The mass density of PMMA is 1.18 g/mL [33]. If the bone cement surface behaved exactly similar to the particle loaded gels, 2.68×10^{-3} g/mL loading would correspond to $(2.68 \times 10^{-3}$ g/1.18g$) \times 100\% = 0.23\%$ of the cement, in w/w. The value is indeed not far from the

observed 1% w/w for the particle loaded PMMA when the impact of inhomogeneous particle distribution on the surface is taken into consideration. More details on this respect are provided in S7 Appendix.

Fig 7. The optical density of the broth in which the *Escherichia coli* ATCC # 25922 cells contacted with the surfaces of PMMA samples, having different amounts of $AgNbO_3$, are incubated. Each data point is average over 3 biological replicas.

Another factor, which contributes to the minor discordance between the antimicrobial efficacy of the antimicrobial particles on gel surface and those loaded into the bone cement, is the dissimilarity of inoculum in the two cases. We used fewer cells on the gel to avoid crowding which causes the merging of the colonies. On the other hand, we used a higher amount of cells on the cement surface to increase our confidence in calling the surface a true antimicrobial. Nonetheless we believe that the experiments on the gel are a good proxy for the microbial proliferation on the antimicrobial surfaces produced by loading antimicrobial particles.

Conclusion

In conclusion, the growth of microbial colonies on the surface of a gel loaded with antimicrobial nanostructured $AgNbO_3$ particles was investigated. It was found that similar to the planktonic case, the impact of sublethal levels of the antimicrobial particles is manifested as prolonged lag phase and a corresponding surface minimum inhibitory concentration (SMIC) could be defined above which the bacterial cells do not form colonies. The SMIC obtained in this way is a good estimator for the required antimicrobial concentration to be loaded into a solid to provide its surface with the antimicrobial property.

References

1. Murray CJL, Ikuta KS, Sharara F, Swetschinski L, Aguilar GR, Gray A, et al. Global burden of bacterial antimicrobial resistance in 2019: a systematic analysis. Lancet. 2022; 399(10325), 629-639. doi: 10.1016/ S0140-6736(21)02724-0

2. Review on Antimicrobial Resistance, "Tackling Drug-resistant Infections Globally: Final Report and Recommendations", London, England, Review on Antimicrobial Resistance, 2016, 1-15.

3. Ahmed SA, Baris E, Go DS, Lofgren H, Osorio-Rodarte I, Thierfelder K. Assessing the Global Economic and Poverty Effects of Antimicrobial Resistance. World Bank Policy Research Working Paper. 2017; 8133, 1-17.

4. Mwangi VN, Madivoli ES, Kangogo M, Wanakai SI, Wando W, Nzilu DM. Antimicrobial surface coating as a pathway to curb resistance: preparation, mode of action and future perspective. Journal of Coatings Technology and Research. 2024; 1-9. doi: 10.1007/s11998-023-00879-z

5. Romano CL, Scarponi S, Gallazi E, Romano D, Drago L. Antibacterial coating of implants in orthopaedics and trauma: a classification proposal in an evolving panorama. Journal of orthopaedic surgery and research. 2015; 10, 3. doi: 10.1186/s13018-015-0294-5

6. Calovi M, Coroneo V, Rossi S. Antibacterial efficiency over time and barrier properties of wood coatings with colloidal silver. Biotechnological products and process engineering. 2023; 107, 5975-5985. doi: 10.1007/s00253-023-12710-1

7. Cazzola M, Barberi J, Ferraris S, Cochis A, Cempura G, Czyrska-Filemonowicz A, et al. Bioactive Titanium Surfaces Enriched with Silver Nanoparticles Through an In Situ Reduction: Looking for a Balance Between Cytocompatibility and Antibacterial Activity. Advanced Engineering Materials. 2023; 25 (2), 1-14. doi: 10.1002/adem.202200883

8. Yildiz T, Durdu S, Ozcan K, Usta M. Characterization and investigation of biological properties of silver nanoparticle-doped hydroxyapatite-based surfaces on zirconium. Scientific reports. 2023; 13 (16733), 1-10. doi: 10.1038/s41598-023-33992-5

9. Pryshchepa O, Pomastowski P, Buszewski B. Silver nanoparticles: Synthesis, investigation techniques, and properties. Advances in Colloid and Interface Science. 2020; 284, 24-26. doi: 10.1016/j.cis.2020.102246

10. Rai M, Yadav A, Gade A. Silver nanoparticles as a new generation of antimicrobials. Biotechnology Advances. 2009; 27, 80. doi: 10.1016/j.biotechadv.2008.09.002

11. Talebpour C, Fani F, Ouellette M, Salimnia H, Alamdari H. Nondegradable Antimicrobial Silver-Based Perovskite. ACS Sustainable Chem. Eng. 2022; 10 (15), 4922-4928. doi: 10.1021/acssuschemeng.1c08181

12. Costerton, J. W. Introduction to biofilm. International journal of antimicrobial agents. 1999; 11, 218-219.

13. Prakash B, Veeregowda BM, Krishnappa G. Biofilms: a survival strategy of bacteria. Current science. 2003; 85 (9), 1302-1303.

14. Jorge P, Magalhães AP, Grainha T, Alves D, Sousa AM, Lopes SP, et al. Antimicrobial resistance three ways: healthcare crisis, major concepts and the relevance of biofilms. FEMS Microbiology Ecology. 2019; 95, 1-9. doi: 10.1093/femsec/fiz115

15. Macia MD, Rojo-Molinero E, Oliver A. Antimicrobial susceptibility testing in biofilm-growing bacteria. Clinical Microbiology and Infection. 2014; 20 (10), 982. doi: 10.1111/1469-0691.12651

16. Alamdari H, Royer S. Mechanochemistry. In: Grander P, Parvulescu VI, Kaliaguine S, editors. Perovskites and related mixed oxides: concepts and applications. John Wiley & Sons; 2015. pp. 30-42. doi: 10.1002/9783527686605

17. CLSI. Methods for Dilution Antimicrobial Susceptibility Tests for Bacteria That Grow Aerobically; CLSI standard M07. Wayne, PA:

18. CLSI. Performance Standards for Antimicrobial Susceptibility Testing; CLSI M100; Wayne, PA, 2020.

19. Bertrand RL. Lag phase is a dynamic, organized, adaptive, and evolvable period that prepares bacteria for cell division. Journal of bacteriology. 2019; 201(7) 10-1128. doi: 10.1128/jb.00697-18

20. Urbaniec J, Xu Y, Hingley-Wilson S, McFadden J. Phenotypic heterogeneity in persisters: a novel ‘hunker’theory of persistence. FEMS Microbiology Reviews. 2022; 46 (1) fuab042. doi: 10.1093/femsre/fuab042

21. You Z, Pearce DJ, Sengupta A, Giomi L. Mono-to multilayer transition in growing bacterial colonies. Physical review letters. 2019; *123*(17), 178001. doi: 10.1103/PhysRevLett.123.178001

22. Su P, Liao C, Roan J, Wang S, Chiou A, Syu W. Bacterial Colony from Two-Dimensional Division to Three-Dimensional Development. PLOS ONE. 2012; 7(11): e48098. doi: 10.1371/journal.pone.0048098

23. Kearns DB. A field guide to bacterial swarming motility. Nature Reviews Microbiology. 2010; 8(9), 634-644. doi: 10.1038/nrmicro2405

24. Wang Q, Suzuki A, Mariconda S, Porwollik S, Harshey RM. Sensing wetness: a new role for the bacterial flagellum. The EMBO journal. 2005; 24(11) 2034-2042. doi: 10.1038/sj.emboj.7600668

25. Harshey RM. Bacterial motility on a surface: many ways to a common goal. Annual Reviews in Microbiology. 2003; 57(1) 249-273. doi: 10.1146/annurev.micro.57.030502.091014

26. Verstraeten N, Braeken K, Debkumari B, Fauvart M, Fransaer J, Vermant J, et al. Living on a surface: swarming and biofilm formation. Trends in microbiology. 2008; 16(10) 496-506. doi: 10.1016/j.tim.2008.07.004

27. Swiecicki J, Sliusarenko O, Weibel DB. From swimming to swarming: Escherichia coli cell motility in two-dimensions. Integrative biology. 2013; 5(12), 1490-1494. doi: 10.1039/c3ib40130h

28. Hobley l, Harkins C, MacPhee CE, Stanley-Wall NR. Giving structure to the biofilm matrix: an overview of individual strategies and emerging common themes. FEMS Microbiol Rev. 2015; 39(5), 649-669 doi: 10.1093/femsre/fuv015

29. You Z. Growth-induced self-organization in bacterial colonies. PhD dissertation, Universiteit Leiden, 2019. Available from: https://scholarlypublications.universiteitleiden.nl/handle/1887/74473

30. Zhang SX, Rawte P, Brown S, Lo S, Siebert H, Pong-Porter S, et al. Evaluation of CLSI agar dilution method and Trek Sensititre broth microdilution panel for determining antimicrobial susceptibility of Streptococcus pneumoniae Journal of clinical microbiology. 2011; 49(2) 704-706. doi: 10.1128/JCM.01622-10

31. Rolfe MD, Rice CJ, Lucchini S, Pin C, Thompson A, Cameron ADS, et al. Lag phase is a distinct growth phase that prepares bacteria for exponential growth and involves transient metal accumulation Journal of bacteriology. 2012; 194(3) 686-701. doi: 10.1128/JB.06112-11

32. Keogh D, Lam LN, Doyle LE, Matysik A, Pavagadhi S, Umashankar S, et al. Extracellular electron transfer powers Enterococcus faecalis biofilm metabolism MBio. 2018; 9(2) 10-1128. doi: 10.1128/mBio.00626-17

33. Dunne N, Tzagiollari A, Sahebalzamani M, Dunne TJ. Acrylic cements for bone fixation in joint replacement. In: Revell P, editor. Joint Replacement Technology (Third Edition). Woodhead Publishing Series in Biomaterials; 2021. pp. 229. doi: 10.1016/B978-0-12-821082-6.00021-2

Supporting information

S1 Appendix. Measuring solid phase growth rate
S2 Appendix. Surface density of $AgNbO_3$ particles on antimicrobial gels
S3 Appendix. Estimating average particle size, volume and mass
S4 Appendix. Determining the equivalent MIC
S5 Appendix. Determining distance between particles
S6 Appendix. Supplementary information for Fig 6
S7 Appendix. Size distribution and density of the antimicrobial particles on the bone cement

Fig 1

Click here to access/download;Figure;Fig 1.tif

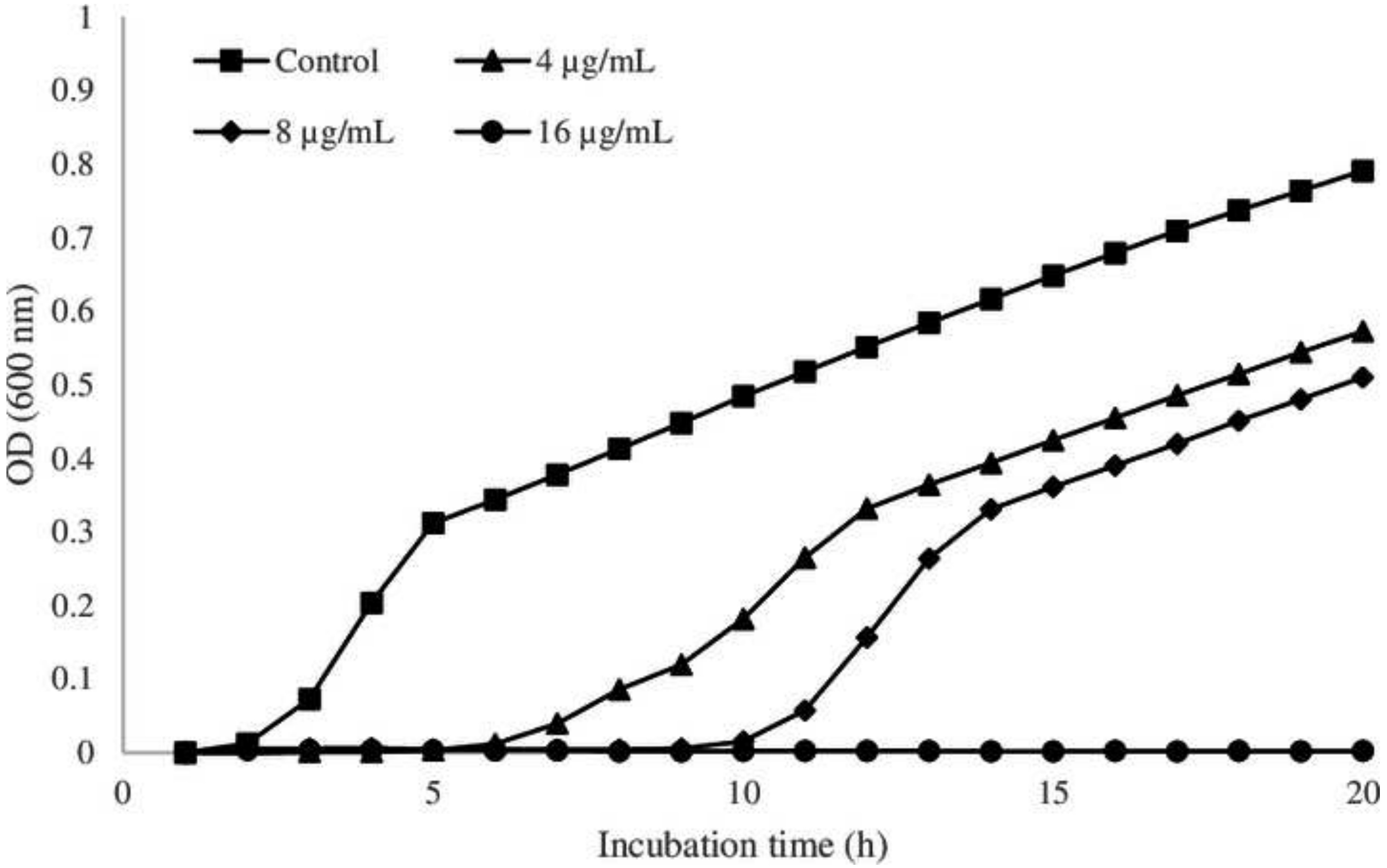

Fig 2

Click here to access/download;Figure;Fig 2.tif

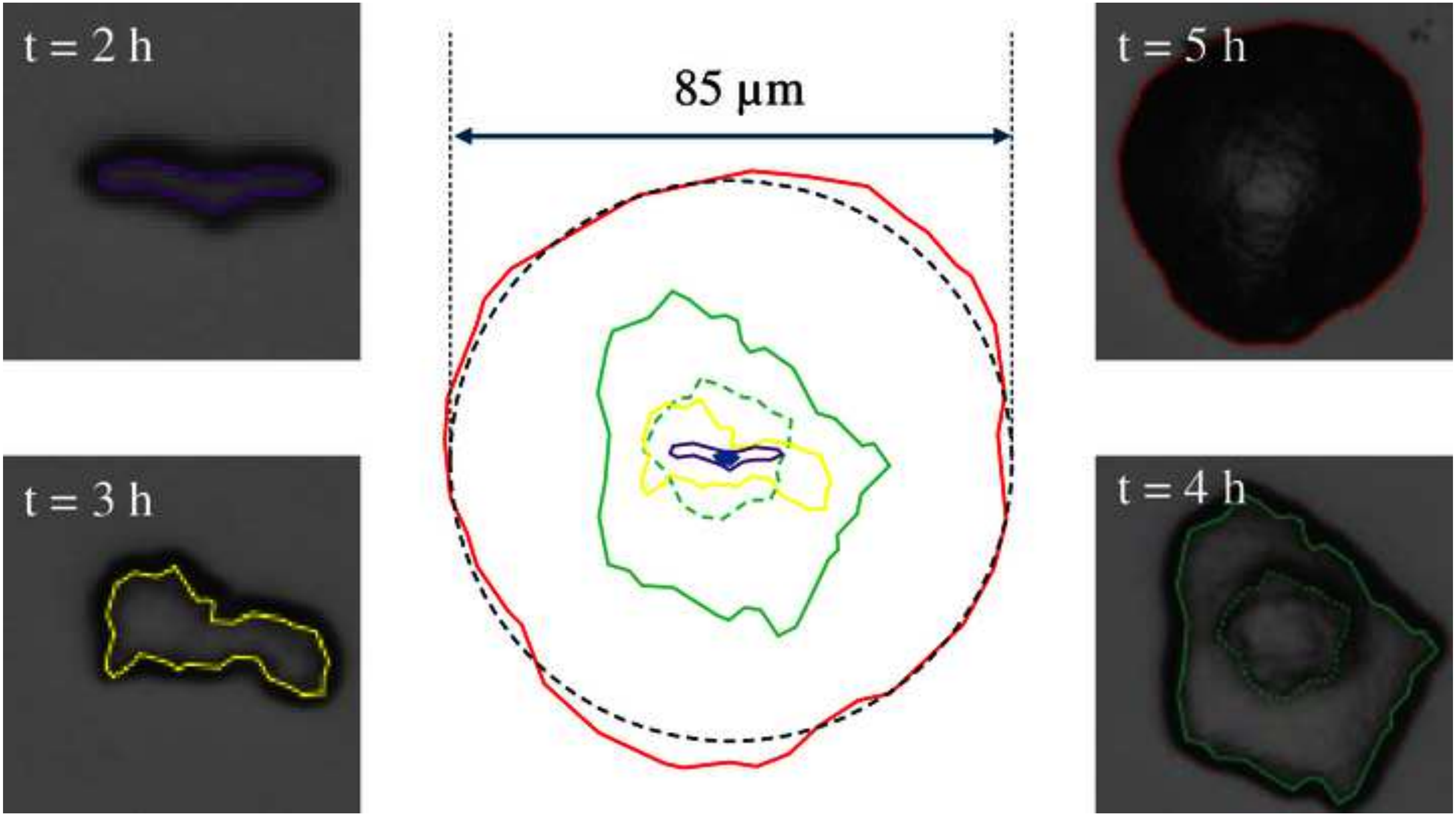

Fig 3

Click here to access/download;Figure;Fig 3.tif

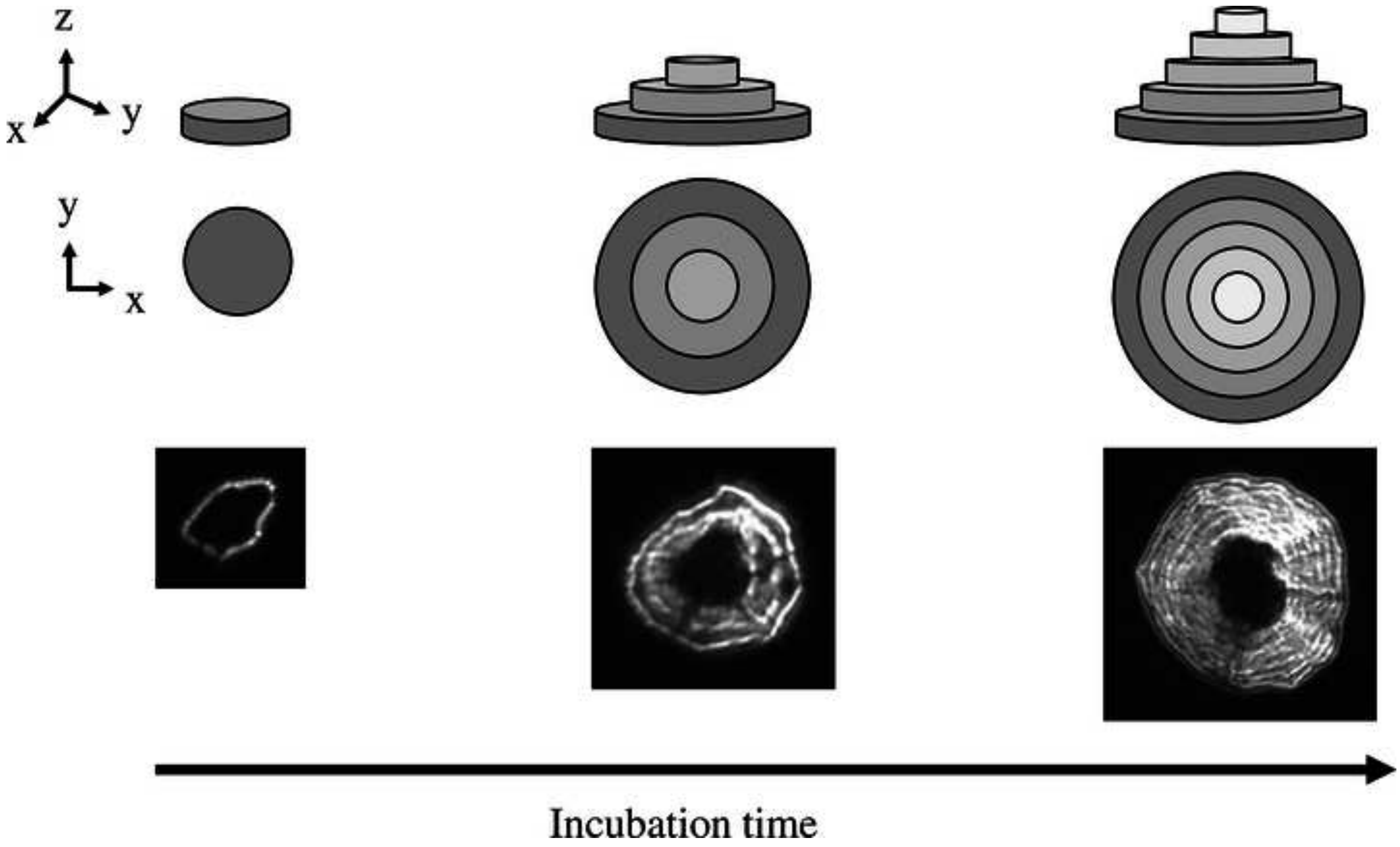

Click here to access/download;Figure;Fig 4.tif

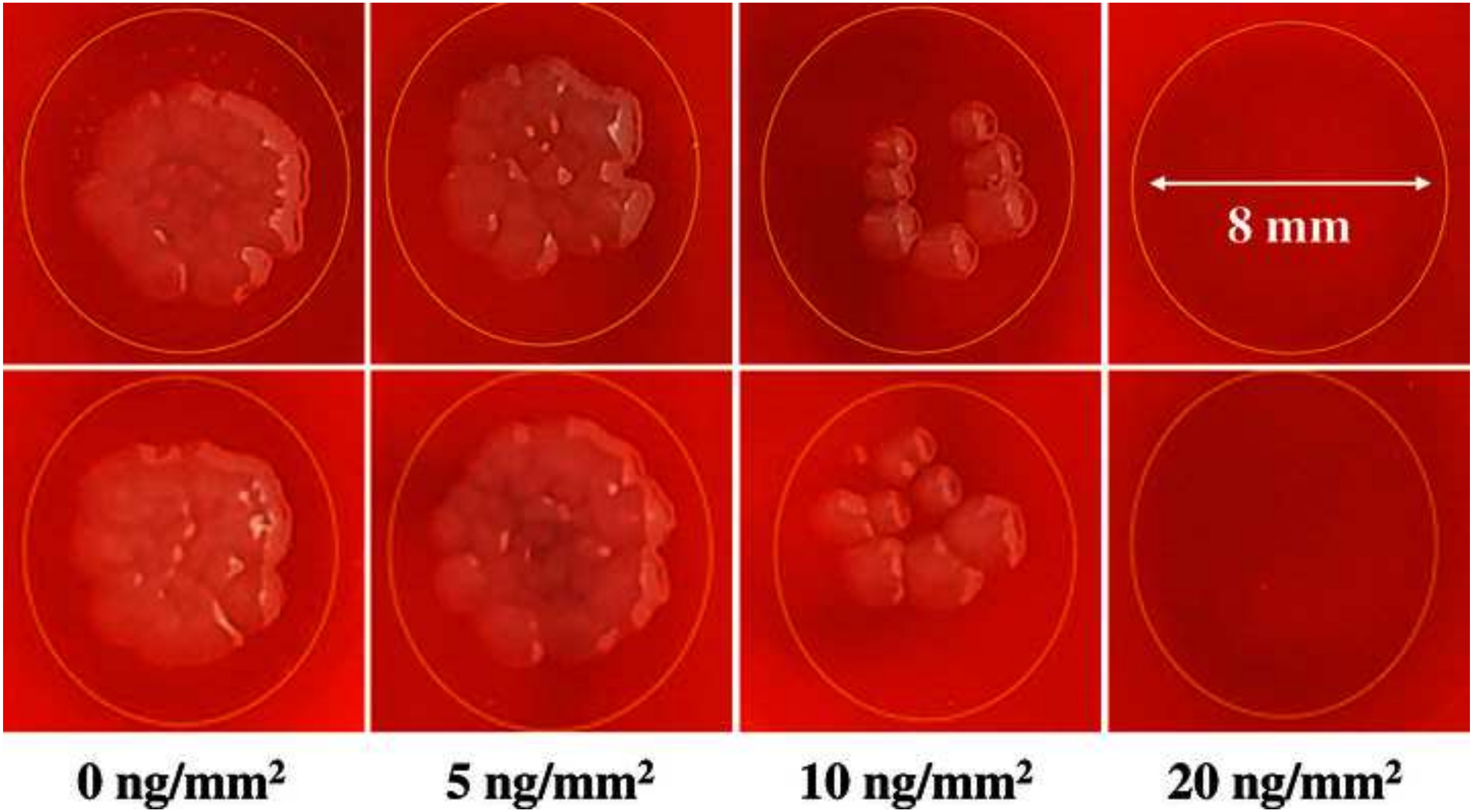

Fig 5

Click here to access/download;Figure;Fig 5.tif

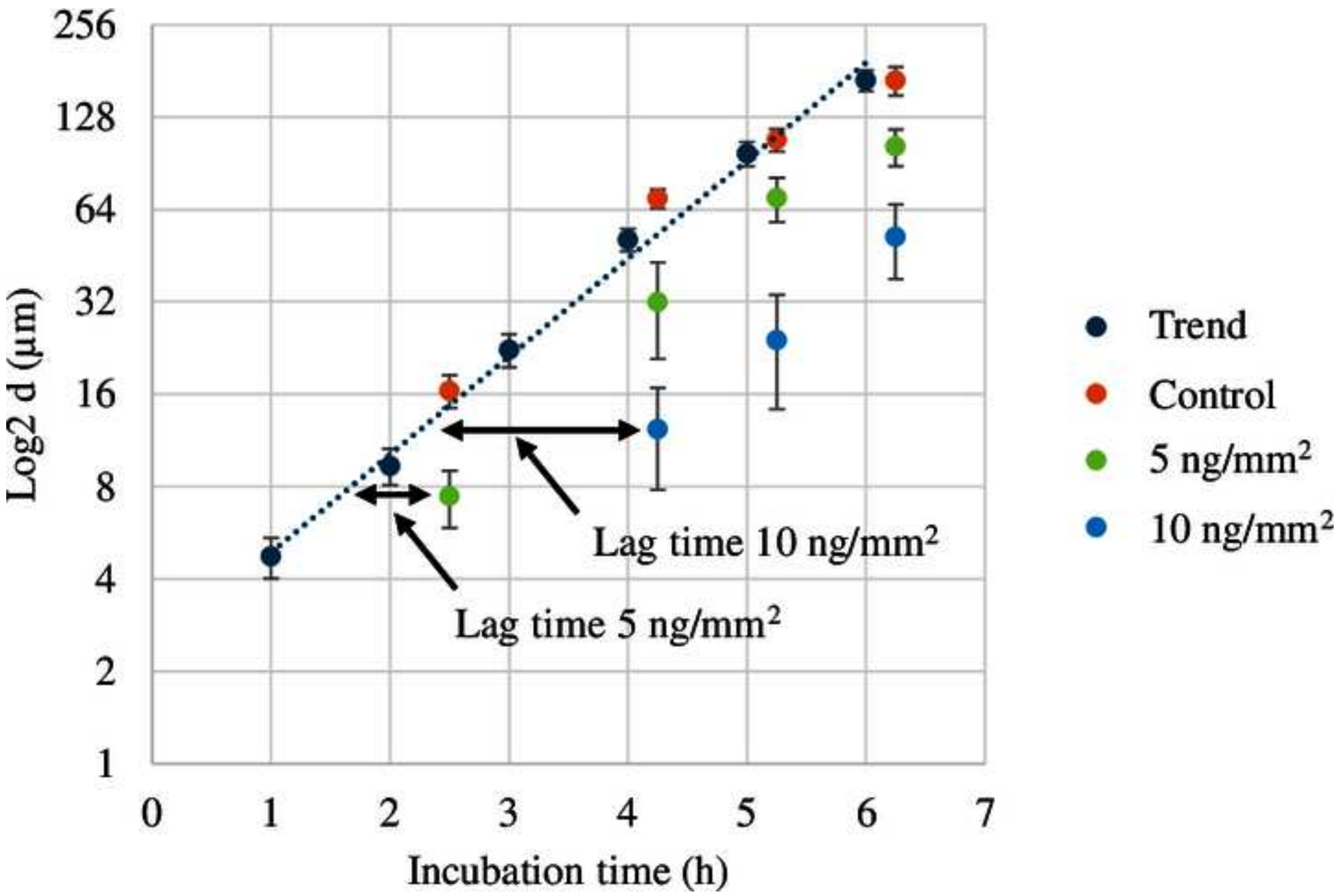

Fig 6

Click here to access/download;Figure;Fig 6.tif

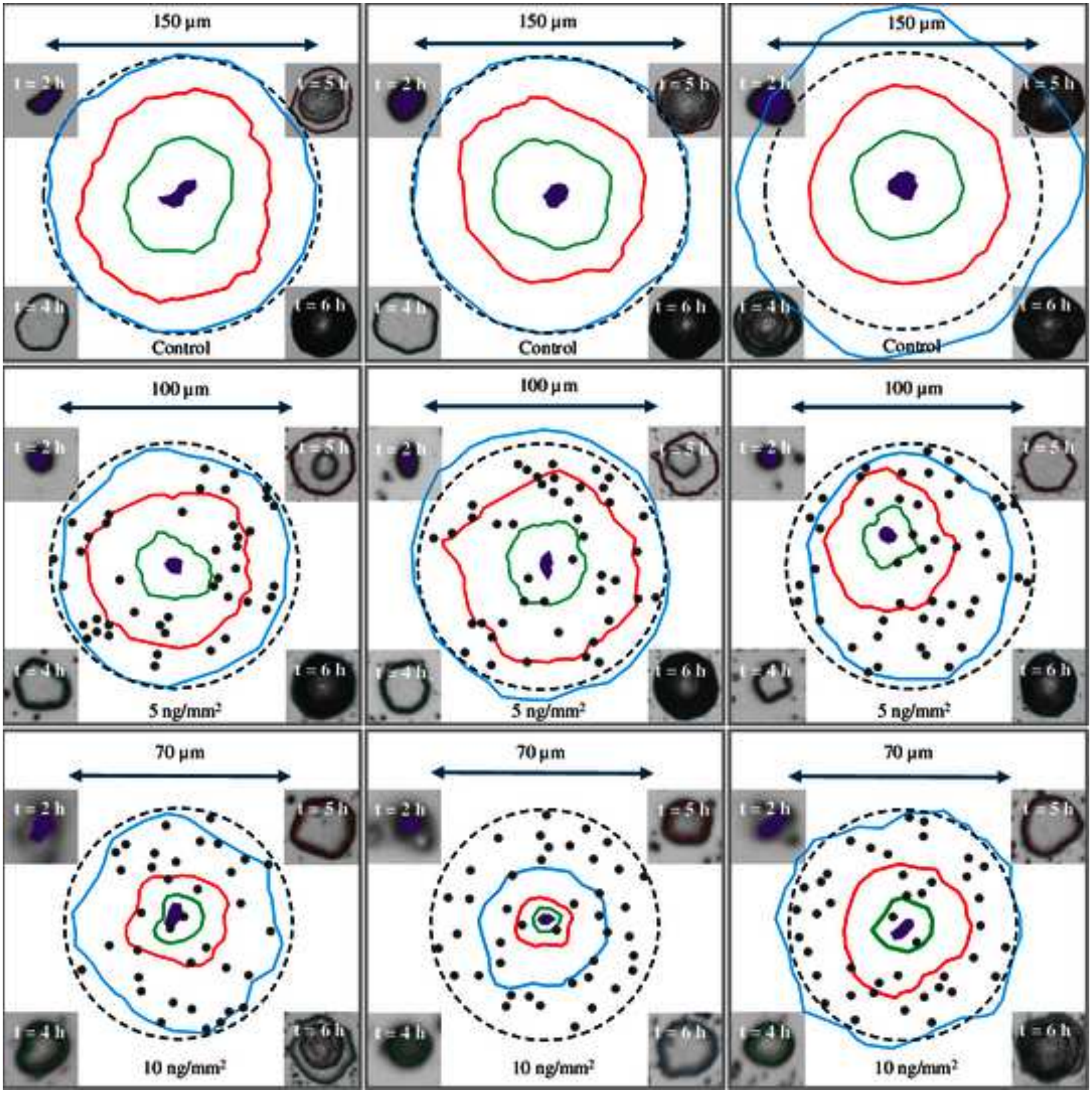

Click here to access/download;Figure;Fig 7.tif

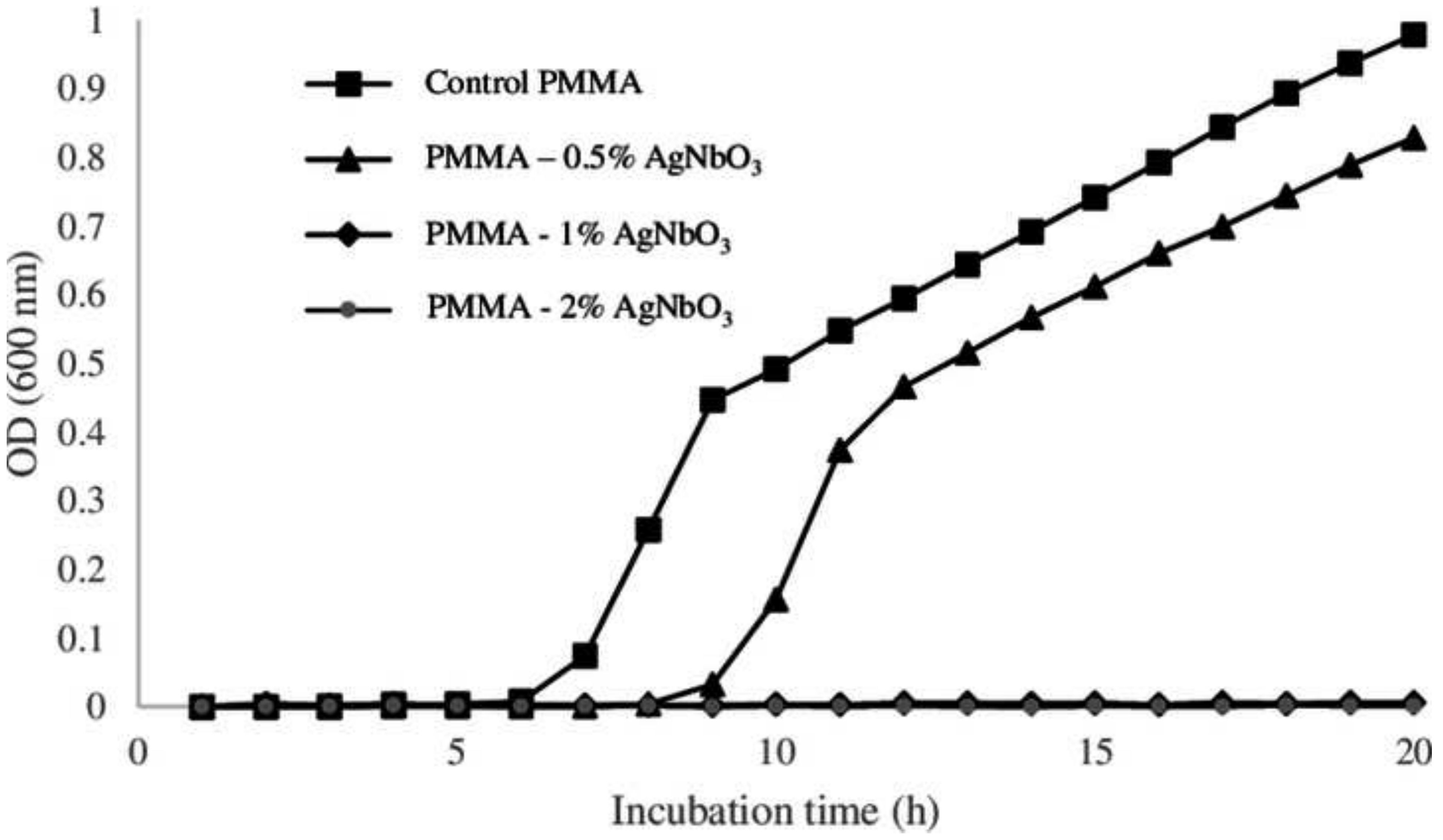

S1 Appendix

Click here to access/download
Supporting Information
S1 Appendix.docx

Click here to access/download
Supporting Information
S2 Appendix.docx

S3 Appendix

Click here to access/download
Supporting Information
S3 Appendix.docx

S4 Appendix

Click here to access/download
Supporting Information
S4 Appendix.docx

Click here to access/download
Supporting Information
S5 Appendix.docx

Click here to access/download
Supporting Information
S6 Appendix.docx

S7 Appendix

Click here to access/download
Supporting Information
S7 Appendix.docx
