# Supplementary material for: Growth dynamics of Escherichia coli cells on a surface having AgNbO3 antimicrobial particles
Source: PLoS One. 2024 Aug 19;19(8):e0305315. doi: 10.1371/journal.pone.0305315 (PMC11332949; doi:10.1371/journal.pone.0305315)
Supplement: Attachment — Submitted filename: revPlosOne.pdf [file pone.0305315.s010.pdf]

The work deals with the study of *E. coli* growth dynamics on a surface with $AgNbO_3$ antimicrobials particles. In this sense, the title is too general because only *E. coli* but not other bacteria were evaluated.

Although the theme and objectives of the work contribute to knowledge in the area of antimicrobial materials, the manuscript have some errors or not clear sentences to correct before being considered for publication in the Journal.

In Methodology section the synthesis of $AgNbO_3$ particles, as part of this work, is described. Then for the characterization the author explain that the particles were characterized in a previous work by Dynamic Light Scattering (DLS), showing an average size of 0.44 µm. Materials of this size are not considered nanomaterial, in general metallic antimicrobial nanoparticles are less than nm. Furthermore, if the synthesis of a material is included in the manuscript, also the characterization results of these materials should be included in both methodology and results. For example antimicrobial properties depend on the characteristic of the nanomaterials which could change (or not) in different synthesis. In this work nanometric size is not evidenced, so all the words with “nano”should be replaced, for example replace “nanoparticles” by ”particles”.

In “Bacterial strain and culture conditions” a long method including centrifuging bacterial cells is described. What is the purpose of this method? If it is based in previous work authors should include a citation. If the aim is obtain a 10^7 bacterial suspension in TSB why do they centrifuge 1 ml of the suspension in TSB and then resuspend in 1 ml TSB?

When describe minimum inhibitory concentration is based in a standardized method (CLSI?) which should be cited.

The subtitle “Seeding microbial cells on solid phase growth media and interrogating their growth” is not clear, should be changed.

In this section, authors describe “The plates were placed inside the incubator” and mention the formation of colonies, however neither the temperature nor the time is described for this assay.

Authors said that disks were sterilized by soaking in 70% isopropanol. However, isopropanol is a disinfectant but not a sterilizing agent, so this sentence should be corrected.

In results of “Growth dynamics of colonies on a surface containing antimicrobial particles” authors report that “the surviving cells on the surface proliferate with an exponential growth rate”. However, in this assay the inhibition growth (bacteriostatic effect) but not the bacterial viability (bactericidal effect) is evaluated, so this sentence should be rewritten.
