# Supplementary material for: Growth dynamics of Escherichia coli cells on a surface having AgNbO3 antimicrobial particles
Source: PLoS One. 2024 Aug 19;19(8):e0305315. doi: 10.1371/journal.pone.0305315 (PMC11332949; doi:10.1371/journal.pone.0305315)
Supplement: Attachment — Submitted filename: Response to Reviewers.docx [file pone.0305315.s011.docx]

Reviewer #1: The manuscript entitled "Bacterial growth dynamics on a surface having a particulate antimicrobial agent' was evaluated. I suggest the manuscript for publishing, before it can be accepted it should revise carefully. First, you should use a better title for reflecting your study. Second, you should provide more information in your abstract section. Your abstract is not very informative. Your introduction and methodology is OK. But your results and discussion needs to improve. It seems you assembled it very fast. You just indicated your results and there is not any comparison with other studies. There are studies such as Antibiotics 2021, 10(1), 87; <https://doi.org/10.3390/antibiotics10010087>, which you can use for improvement of your manuscript. So, I highly recommend to do a major revision on your manuscript , provide more details , specially in your results and discussion section, for reconsideration. Also, provide your statistical analysis which you used in this study.

Thank you very much for the comments. The title was modified to the following:

“Growth dynamics of *Escherichia coli* cells on a surface having AgNbO_3_ antimicrobial particles”

We added some description into the abstract relating higher surface concentration of particles to delayed bacteria growth or lack thereof.

We have added Fig 7 to the manuscript to support our added discussion on bacteria growth after induced lag phase at sub-MIC concentrations of antimicrobial particles. We related this topic to the “bistable effect” described in your suggested reference. This discussion is furthermore supported by a new S7 Appendix.

Finally, we modified Table 1 to include our statistical analysis, we have used the two-sample T-test for this purpose.

Reviewer #2: In this manuscript, the authors investigated the morphological dynamics of microbial cell proliferation on an antimicrobial surface using E. coli on the surface of a gel incorporating AgNbO₃ particles. The manuscript is well written; however, there are some concerns as follows:

1. Regarding the last paragraph of the Introduction, "It is worth mentioning that incorporation of the right amount of AgNbO₃ nanoparticles in PMMA bone cement is expected to confer permanent antibacterial property to the cement due to its extremely limited corrosion/dissolution rate, contrary to the incorporation of conventional antibiotics such as gentamicin," this hypothesis is unclear as the manuscript does not specifically verify the "permanent antibacterial property."

This hypothesis was verified in one of our previous publications (Published after the initial submission of this manuscript), where a PMMA sample mixed with 1 % AgNbO_3_ particles (w/w) AgNbO_3_ showed persistence of antibacterial properties over a four-month period with no reduction in its potency. We accordingly add the reference to the sentence:

“It is worth mentioning that incorporation of the right amount of AgNbO_3_ nanoparticles in PMMA bone cement is expected to confer permanent antibacterial property to the cement due to its extremely limited corrosion/dissolution rate, contrary to the incorporation of conventional antibiotics such as gentamicin, as verified in our previous publication [16]. There, we demonstrated the persistence of antibacterial properties for a PMMA sample mixed with 1 % AgNbO_3_ particles (w/w) over a four-month period with no reduction in its potency when kept in water.”

2. In section 2.5, “Using imageJ software, the microscopic images were analyzed for extracting the characteristic features of three representative colonies within one spot for each condition, and the average size of the 20 representative colonies from the two spots for each condition.” It’s not comprehensible to relate “20 representative colonies” to “three representative colonies within one spot”. And the result in Table 1 showed “the average size of 10 representative colonies”.

Thank you for your comment. To clarify, 20 representative colonies were selected for calculating the average size of colonies given a condition of a determined concentrations of the antimicrobial particles and incubation time. Of these 20 colonies, only three were selected for extracting characteristic features. In the latter case, we find that for discussion three representative colonies are sufficient to present in Fig 6. Accordingly, we applied the appropriate correction for further clarification of the text:

“The area of 20 colonies, from the images of the two spots for each condition, was selected using ImageJ software. Each condition represents a given combination of antimicrobial particle concentration and incubation time. Then, the average area was calculated for determining the average colony size for that given condition. For each condition, three representative colonies were selected for extracting characteristic features.”

3. Did you polish the resin specimens incorporating nanoparticles in the methods section 2.6? If so, please specify the type of polishing paper used and the grit sizes used for polishing. This information should also be included in S7 Appendix.

For PMMA specimens, polishing was deliberately not performed for the antimicrobial testing, since the established application of PMMA in orthopedic operations involves pouring the mixture in situ and letting it solidify without any further treatment. Therefore, our attempt in preparing a “true antimicrobial surface” with an aim in orthopedic applications should follow the similar preparation procedures.

That said, for microscopic observations, polishing is a necessity and was performed for the specimens used. The procedure was included in what was originally S7 Appendix (Now S8 Appendix).

4. There was no icon of “20 μg/mL” in Fig.1.

Thank you for your comment. We assume by 20 μg/mL you meant 32 μg/mL and proceed to answer as below. Please let us know if other specific action is required to satisfy the comment:

Indeed, we recorded the OD_600_ of bacteria exposed to concentrations of 0, 4, 8, 16 and 32 μg/mL of AgNbO_3_ particles. Because there was no bacteria growth when exposed to either of 16 or 32 μg/mL of AgNbO_3_, we omitted reporting of the 32 μg/mL condition in Fig 1 and added the following sentence in the paragraph preceding Fig 1:

“The same lack of increase in OD_600_ was seen for the case of AgNbO_3_ concentration of 32 μg/mL, which was not included in Fig 1 due to overcrowding.”

5. In section 3.2, equation (1) is is not accessible.

Thank you for letting us know.

Equation 1 describes the relation between a circle’s Area and its diameter (d=2*sqrt(Area/Pi). We re-wrote the equation in the manuscript, but all equations throughout the manuscript body and supporting appendices were written using Microsoft equation and should be visible in PDF file. We will double-check the visibility of the equations in revised PDF version.

6. In section 3.2, there was no explanation about how to relate the increase factor to division cycles.

We modified the sentence in question to the following:

“After an extra hour of incubation, the average colony area increases to 2044 μm2, which is an increase by a factor of 5.2. By taking the log base 2 of 5.2, we get ~2.4 division cycles.”

7. In section 3.3, “However, first report our observation with the growth behavior of bacteria on the surface of gels containing sub-MIC surface concentration of antimicrobial particles.” The sentence is not comprehensible.

We corrected the sentence. Thank you.

“However, we first report our observation with the growth behavior of bacteria on the surface of gels containing sub-MIC surface concentration of antimicrobial particles.”

8. In section 3.3, “For example, at ¼ and ½ the surface MIC, i.e. gels with 5 ng/mm2 and 10 ng/mm2 nanoparticles, the lag phases are 1 and 2 h respectively (Fig 1).” The reference should be Fig. 5.

Thank you very much. We corrected the sentence:

“For example, at ¼ and ½ the surface MIC, i.e. gels with 5 ng/mm^2^ and 10 ng/mm^2^ nanoparticles, the lag phases are 1 and 2 h respectively (Fig 5).”

9. In Fig A-C in S6 Appendix, why the author exhibited the images after 5 h of incubation, not 6 h?

To address this question we included the following explanation at the beginning of section 3.3 of the manuscript:

“The presented images are of after 5 h of incubation specifically because at this time point the colonies are comparatively large but their characteristic morphology has not been perturbed due to formation of multiple layers and extracellular matrix. In addition, at the next point (6 h of incubation) some colonies merge with their neighbors.”

10. Regarding the statement in the results section 3.4, "The predictive capability of our findings on the gel surface MIC was tested by assessing the antimicrobial level of a composite material," evaluating the growth of planktonic bacteria in the presence of nanoparticles, as mentioned in section 2.3, is beneficial for assessing the antibacterial properties of AgNbO3 itself. However, although evaluating colony formation on an agar surface with nanoparticles, as in section 2.4, is appropriate, it is not suitable for predicting bacterial growth on materials such as PMMA. The surface characteristics of the agar medium differ from those of PMMA, and therefore, adding nanoparticles to PMMA and assessing bacterial growth on its surface cannot be considered “predictive”.

We corrected the sentence as the following:

“Our findings on the gel surface MIC was used as an insight for estimating the antimicrobial level of a composite material.”

Reviewer #3: The work deals with the study of E. coli growth dynamics on a surface with AgNbO_3_ antimicrobials particles. In this sense, the title is too general because only E. coli but not other bacteria were evaluated.
Although the theme and objectives of the work contribute to knowledge in the area of antimicrobial materials, the manuscript have some errors or not clear sentences to correct before being considered for publication in the Journal. See attached file.

**Questions from “PONE-D-24-21073.pdf-With comments.pdf”**

1. Your title is very general, Please specify the name of your bacterium, which you used also, indicate your particulate you used in the study.

Thank you for this comment.

The title was modified to the following:

“Growth dynamics of *Escherichia coli* cells on a surface having AgNbO_3_ antimicrobial particles”

2. Your abstract is very concise, please add more detailed information in your abstract.

We added some description into the abstract relating higher surface concentration of particles to delayed bacteria growth or lack thereof.

3. Please provide more data and improve your results and discussion section.

We have added Fig 7 to the manuscript to support our added discussion on bacteria growth after induced lag phase at sub-MIC concentrations of antimicrobial particles. This discussion is furthermore supported by a new S7 Appendix. We modified Table 1 to include our statistical analysis, we have used the two-sample T-test for this purpose.

**Questions from “revPlosOne.pdf”**

1. The work deals with the study of E. coli growth dynamics on a surface with AgNbO_3_ antimicrobials particles. In this sense, the title is too general because only E. coli but not other bacteria were evaluated.

Thank you for this comment.

The title was modified to the following:

“Growth dynamics of *Escherichia coli* cells on a surface having AgNbO_3_ antimicrobial particles”

2. Although the theme and objectives of the work contribute to knowledge in the area of antimicrobial materials, the manuscript have some errors or not clear sentences to correct before being considered for publication in the Journal. In Methodology section the synthesis of AgNbO_3_ particles, as part of this work, is described. Then for the characterization the author explain that the particles were characterized in a previous work by Dynamic Light Scattering (DLS), showing an average size of 0.44 μm. Materials of this size are not considered nanomaterial, in general metallic antimicrobial nanoparticles are less than nm. Furthermore, if the synthesis of a material is included in the manuscript, also the characterization results of these materials should be included in both methodology and results. For example antimicrobial properties depend on the characteristic of the nanomaterials which could change (or not) in different synthesis. In this work nanometric size is not evidenced, so all the words with “nano” should be replaced, for example replace “nanoparticles” by ”particles”.

Thank you for the comment. The reason for which we initially used the term Nano is because the crystallite size of the particles, as measured by X-ray diffraction using Scherrer equation in our past publication, is in the range of nanometer (10-15 nm) [1]. According to the generally accepted definition (Nanotechnology is the branch that comprises the synthesis, engineering, and utilization of materials whose size ranges from 1 to 100 nm, known as nanomaterials), this material is considered as nano-structured material. An example of this definition can be found in a review by Hasan S. [2].

However, due to the agglomeration of these nano-sized crystallites, the individual particles could be much larger than 100 nm (as evidenced by our particle size distribution analysis by dynamic light scattering in our past publication [1]).

So, to avoid any confusion, we accept the recommendation and any word alluding to the particles being “nano” has been removed or replaced both in the manuscript and supporting information.

1. Talebpour C, Fani F, Ouellette M, Salimnia H, Alamdari H. Nondegradable Antimicrobial Silver-Based Perovskite. ACS Sustainable Chem. Eng. 2022; 10 (15), 4922-4928. doi: 10.1021/acssuschemeng.1c08181

2. Hasan S. Review on Nanoparticles: Their synthesis and types. Research Journal of Recent Sciences. 2015; 4 (ISC-2014), 9-10.

4. In “Bacterial strain and culture conditions” a long method including centrifuging bacterial cells is described. What is the purpose of this method? If it is based in previous work authors should include a citation. If the aim is obtain a 10^7^ bacterial suspension in TSB why do they centrifuge 1 ml of the suspension in TSB and then resuspend in 1 ml TSB?

Centrifugation was performed before resuspension to wash out debris.

We made the following addition to section 2.2 of the manuscript:

“Then, a 1 mL culture was transferred to 2 mL autoclaved tubes and centrifuged at 8000 rpm for 8 min in a microcentrifuge to wash out debris, which may appear as artifacts during imaging.”

5. When describe minimum inhibitory concentration is based in a standardized method (CLSI?) which should be cited.

The method of determining minimum inhibitory concentration of an antimicrobial by measuring OD_600_ is not standardized under CLSI guidelines. The method can be rather considered as an experimental model for characterizing the activity of an antimicrobial. An example in the literature using this model to demonstrate the MIC of silver-based antimicrobials on *Escherichia coli* is presented by Haque et al. [3].

We accordingly added this reference to the manuscript in section 2.3 as follows:

“The minimum inhibitory concentration (MIC) was determined as the concentration of particles for which no growth was observed after 24 h of incubation. This approach was adopted from the methodology presented by Haque et al. [18].”

3. Haque MA, Imamura R, Brown GA, Krishnamurthi VR, Niyonshuti II, Marcelle T, et al. An experiment-based model quantifying antimicrobial activity of silver nanoparticles on *Escherichia coli*. RSC Advances/ 2017; 7, 56173-56177. doi: 10.1039/c7ra10495b

6. The subtitle “Seeding microbial cells on solid phase growth media and interrogating their growth” is not clear, should be changed. In this section, authors describe “The plates were placed inside the incubator” and mention the formation of colonies, however neither the temperature nor the time is described for this assay.

Thank you for this comment. The title of section 2.5 was changed to:

“Plating microbial cells and interrogating the morphological evolution and size of the resulting microbial cell colonies”

We added the following sentence to section 2.5:

“The plates were placed inside the incubator at 37 ^°^C for 6 h.”

8. Authors said that disks were sterilized by soaking in 70% isopropanol. However, isopropanol is a disinfectant but not a sterilizing agent, so this sentence should be corrected.

Thank you for the remark. The sentence was modified accordingly:

“Briefly, three replicates of each disk were disinfected by soaking in 70% isopropanol for one min, washing in sterile water for 2 min, and allowing them to air dry.”

9. In results of “Growth dynamics of colonies on a surface containing antimicrobial particles” authors report that “the surviving cells on the surface proliferate with an exponential growth rate”. However, in this assay the inhibition growth (bacteriostatic effect) but not the bacterial viability (bactericidal effect) is evaluated, so this sentence should be rewritten.

This is correct. We modified the terminology in two sentences of section 3.3:

“15% of the dispensed cells did not proliferate on the gel having 5 ng/mm^2^ of AgNbO_3_ particles.”

“Finally, and similarly to planktonic cells (Fig 1), the cells whose growth was not inhibited, after a lag time, proliferate with an exponential growth rate, similar to the case of no antimicrobial particles.”
